# Multimaterial 3D printed self-locking thick-panel origami metamaterials

**DOI:** 10.1038/s41467-023-37343-w

**Published:** 2023-03-23

**Authors:** Haitao Ye, Qingjiang Liu, Jianxiang Cheng, Honggeng Li, Bingcong Jian, Rong Wang, Zechu Sun, Yang Lu, Qi Ge

**Affiliations:** 1 Shenzhen Key Laboratory of Soft Mechanics & Smart Manufacturing, Southern University of Science and Technology, Shenzhen, 518055 China; 2 Department of Mechanical and Energy Engineering, Southern University of Science and Technology, Shenzhen, 518055 China; 3 Department of Mechanical Engineering, City University of Hong Kong, Kowloon, Hong Kong SAR China; 4 Nano-Manufacturing Laboratory (NML), Shenzhen Research Institute of City University of Hong Kong, Shenzhen, China; 5 Department of Mechanical Engineering, The University of Hong Kong, Pokfulam, Hong Kong SAR China

**Keywords:** Mechanical engineering, Mechanical properties

## Abstract

Thick-panel origami has shown great potential in engineering applications. However, the thick-panel origami created by current design methods cannot be readily adopted to structural applications due to the inefficient manufacturing methods. Here, we report a design and manufacturing strategy for creating thick-panel origami structures with excellent foldability and capability of withstanding cyclic loading. We directly print thick-panel origami through a single fused deposition modeling (FDM) multimaterial 3D printer following a wrapping-based fabrication strategy where the rigid panels are wrapped and connected by highly stretchable soft parts. Through stacking two thick-panel origami panels into a predetermined configuration, we develop a 3D self-locking thick-panel origami structure that deforms by following a push-to-pull mode enabling the origami structure to support a load over 11000 times of its own weight and sustain more than 100 cycles of 40% compressive strain. After optimizing geometric parameters through a self-built theoretical model, we demonstrate that the mechanical response of the self-locking thick-panel origami structure is highly programmable, and such multi-layer origami structure can have a substantially improved impact energy absorption for various structural applications.

## Abstract (web summary)

Thick panel origami holds great potential in engineering structures, but conventional fabrication processes limit their design and applications. Here the authors report a multimaterial 3D printing-based design and fabrication strategy for thick-panel origami structures with push-to-pull deformation.

## Introduction

Mechanical metamaterials are artificial materials with mechanical properties dominated by their geometry instead of their composition^1–4^. Because mechanical metamaterials exhibit exceptional mechanical properties and functionalities that cannot be obtained in a bulk material, they have arisen intensive research interests and found applications in various fields^5–10^. Recently, researchers have shown great interests in the origami-based metamaterials^11–13^, which exhibit infinite possibilities for geometry transformation from 2D surface to 3D structure to achieve unconventional properties such as negative Poisson’s ratio^14,15^, tunable/graded mechanical properties^16–19^ and multi-stability^20–22^.

Rigid-foldable origami or rigid origami^23^ is a subset of origami, where facets that are typically rigid panels rotate around predetermined hinges without any tension-bend deformation during continuous folding process^24^. Therefore, rigid-foldable origami can be regarded as a deployment mechanism with stiff panels and hinges, which have advantages in various engineering applications^25^. In general, rigid-foldable origami patterns are created from idealized models that treat the facets as having zero thickness. In order to make rigid-foldable origami more applicable to engineering applications, researchers have proposed ideas of developing thick-panel origami to enhance stiffness of origami panels^26^. To date, many techniques have been proposed for accommodating thickness in the folding motion of nonzero thickness panels, i.e. tapered panels^23^, offset panels^27^, hinge shifting^28,29^, and so on. However, most of the thick-panel origami structures were manufactured by tedious manual assembly processes such as using adhesive tapes^27,30^ or rolling hinges^31,32^ to connect thick panels, which are not favorable for real engineering applications.

Three-dimensional printing (3D) printing is an emerging advanced manufacturing technology that creates complex 3D objects in free form^33^. The fused deposition modeling (FDM) 3D printing forms 3D structures by melting thermoplastic filament and extruding it through the printing nozzle to generate patterns for each layer according to predetermined tool paths. Compared with other 3D printing technologies, FDM 3D printing is the most widely used one due to its simplicity, inexpensiveness, and compatibility with various engineering plastics. Many attempts have been made to fabricate origami structures through FDM 3D printing^34–37^. However, the mainstream single material printing capability places users in a dilemma: origami structures printed with rigid material can withstand loads but are not foldable; instead, soft material imparts foldability to the printed origami structures by sacrificing load capacity. The capability of 3D printing soft and rigid materials into one structure is desired for fabricating thick-panel origami structures. Wagner et al.^38^ reported FDM 3D printing approaches to print aramid fibers and polyamide hinges which could fold ~10^6^ times, while its direct application to origami is yet to be demonstrated. Faber et al.^39^ fabricated a bioinspired spring origami which achieves rigid origami folding and variable stiffness by printing stiff polymer facets (acrylonitrile butadiene styrene - ABS) onto rubberlike substrates (thermoplastic polyurethane - TPU). Nevertheless, the weak interfacial bonding between ABS and TPU may lead to delamination between them, and cause structural failure during folding-unfolding process of the printed origami structure. Moreover, these foldable origami structures usually could not support loads as small perturbations may collapse the structure along the same folding-unfolding path.

Here, we report a FDM multimaterial 3D printing-based manufacturing and design approach for creating thick-panel origami structures which are not only foldable but also capable of supporting heavy load and sustaining cyclic loads under large compressive strain. We fabricate the thick-panel origami structures on an FDM multimaterial 3D printer following a special fabrication strategy where the rigid panels are wrapped and connected by highly stretchable soft parts. This fabrication strategy enables the thick-panel origami to fold more than 100 times without debonding. Through stacking two pieces of thick-panel origami panels into a predetermined configuration, we develop self-locking Miura-origami units which are foldable and capable of bearing compression through the unconventional push-to-pull (P2P) deformation mode that converts the vertical compressive force (push) to the stretch of horizontal soft hinges (pull), and makes the self-locking origami structure able to support the load which is over 11000 times of its own weight and sustain more than 100 cycles of 40% compressive strain. Moreover, we build a theoretical model to guide the design of this self-locking Miura-origami structure to exhibit P2P deformation mode rather than bucking deformation mode. The special multimaterial 3D printing strategy combined with the P2P deformation mode endow self-locking Miura-origami with programmable mechanical properties to support heavy static load as well as excellent capability of absorbing impact energy. A three-layer origami structure is capable of reducing the peak impact force from 11897.41 N to 3314.37 N from a 72 J impact (72.1% reduction in the peak impact force). The manufacturing and design approach proposed in this work provide new insights for implementing origami in practical engineering applications.

## Results and discussion

### Multimaterial 3D printing of thick-panel origami

**Fig. 1:**
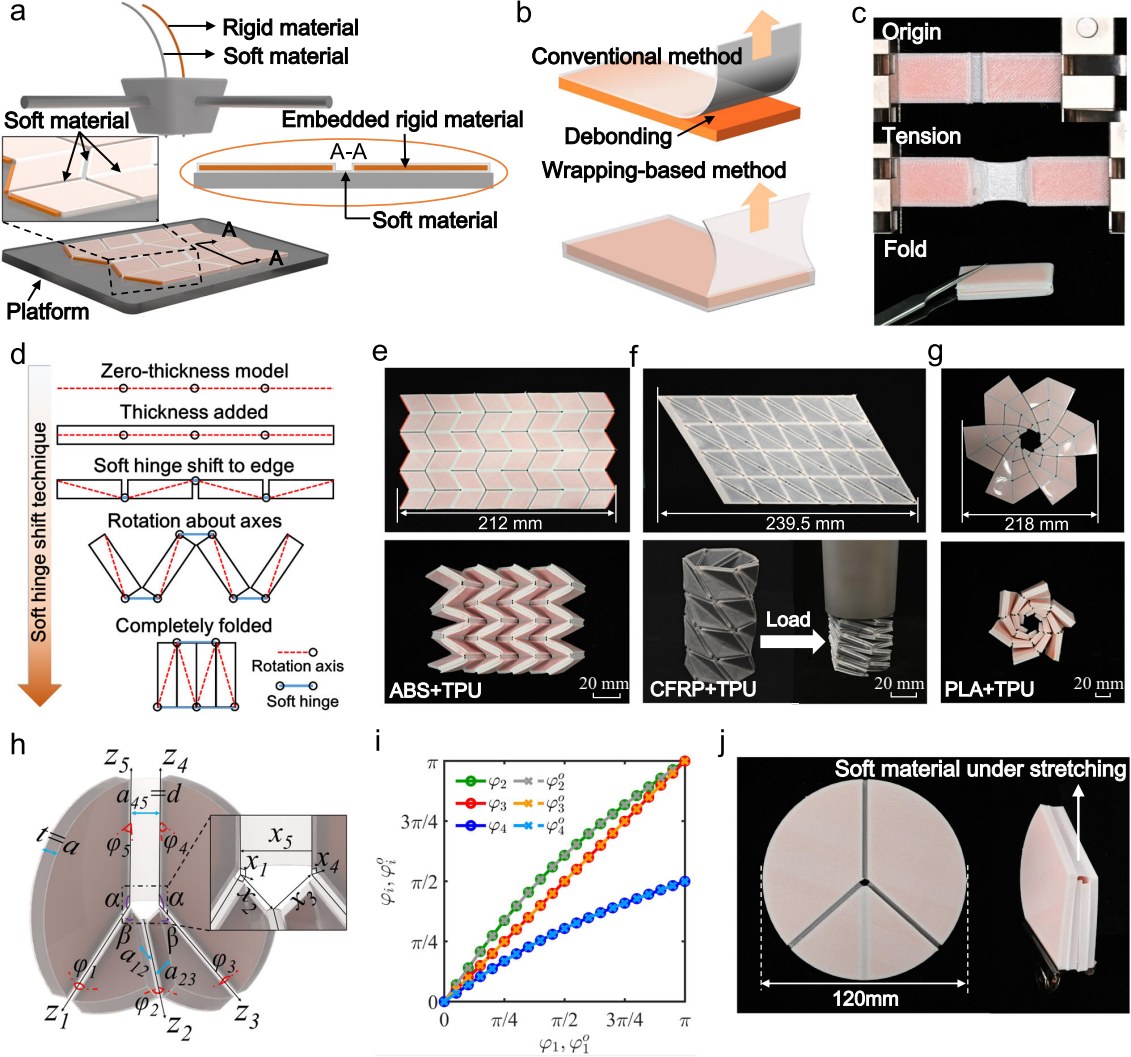
Fabrication and design of thick-panel origami. **a** Illustration of thick-panel origami fabrication through FDM multimaterial 3D printing. **b** Comparison on the interfacial property of multimaterial 3D printed samples by conventional and wrapping-based strategies. **c** Snapshots of wrapping-based hinge under tension and fold. **d** Concept of soft hinge shift technique. **e**–**g** Demonstrations of 3D printed thick-panel origami with Miura, Kresling, flasher pattern, whose rigid component composed by ABS, CFRP, PLA, respectively (scale bars = 20 mm). **h** Illustration of a thickened plane-symmetric single-vertex four-crease origami model. **i** Relationships between dihedral angles for zero-thickness and thickened single-vertex four-crease origami model. **j** Demonstration on the foldability of 3D printed thickened plane-symmetric single-vertex four-crease origami.

Figure 1a illustrates the rigid-soft coupled multimaterial 3D printing strategy via a commercial FDM multimaterial 3D printer (Ultimaker S5, Netherlands) to create thick-panel origami where rigid panels and soft hinges are firmly bonded. In this multimaterial 3D printing strategy, a rigid panel consists of a rigid material (i.e., polylactic acid -PLA) as core wrapped by soft material (i.e., TPU) as skin; the soft material is continuous in both soft hinges and rigid panels so that the two parts can be firmly bonded. As illustrated in Fig. 1b, the conventional FDM multimaterial 3D printing method prints thick-panel origami structure through simply depositing soft parts onto the rigid substrate, which would easily lead to debonding between the soft and rigid materials at the interface. In contrast, the wrapping-based 3D printing strategy proposed in this work could effectively avoid delamination between rigid and soft components even when a printed rigid-soft coupled structure is under large tension. As shown in Supplementary Fig. 1, the printed Miura-origami sheet with rigid PLA sheets wrapped by TPU skins can sustain more than 100 cycles of folding-unfolding, and no fracture was founded after the cyclic test. In comparison, Supplementary Fig. 2 shows that the delamination can be easily found on the Miura-origami sheet that has the same design but was printed by simply depositing the rigid PLA panels onto the TPU substrates. Figure 1c demonstrates the robust interfacial bonding between the soft and rigid parts, which allows the printed hinge to sustain large tension and achieve rigid folding without obvious fracture. Supplementary Fig. 3 also presents the effect of printing parameters (such as raster angle and layer thickness) on the mechanical property of printed hinges. It should be noted that the proposed rigid-soft coupled multimaterial 3D printing strategy is not material-dependent, and applicable to couple various rigid (such as PLA, ABS, carbon fiber reinforced polymer – CRRP) and soft materials (such as TPU, and other thermoplastic elastomers). As shown in Supplementary Fig. 4, the printed hinges with different constituent materials exhibit similar mechanical behavior. This multimaterial 3D printing strategy successfully solves the weak interfacial bonding issue occurring in FDM multimaterial 3D printing (Supplementary Figs. 2 and 5), and therefore can be adopted to fabricate thick-panel origami structures consisting of rigid panels and soft hinges.

In traditional zero-thickness origami patterns, a 3D origami structure is created by folding the “mountain” and “valley” creases on origami pattern. However, once the thickness is added to origami pattern, preserving kinematics and assuring non-self-intersection become critical during the folding process. In Fig. 1d, we present a soft hinge shift technique to accommodate the thickness of panels and avoid interference between thick panels by shifting the location of the rotation axes away from the neutral plane based on the “mountain” and “valley” creases design. Different from other axis shift techniques for origami folding^28,40^, the soft hinge made of highly stretchable soft material is stretched to accommodate the panel thickness. In Fig. 1e–g, we demonstrate the generality of this wrapping-based multimaterial 3D printing strategy for thick-panel origami design and fabrication. It can fabricate thick-panel origami with different origami patterns, i.e. Miura origami (Fig. 1e), Kresling origami (Fig. 1f), and origami flasher (Fig. 1g) where the rigid parts are printed with different materials, i.e. ABS (Fig. 1e), CFRP (Fig. 1f), PLA (Fig. 1g). This wrapping-based strategy enables the flat thick-panel origami to be directly printed in a single step without tedious assembly processes, which greatly saves the fabrication time and improving the manufacturing accuracy.

This high stretchability of TPU imparts the foldability to 3D printed thick-panel origami. To verify this foldability, we modify a kinematic model based on D-H convention (Supplementary Fig. 6a) for thick-panel origami^41,42^ by taking account of the high stretchability of the soft hinges. As shown in Fig. 1h, the plane-symmetric four-crease single vertex origami model is employed to verify the foldability of the corresponding nonzero thickness origami pattern. In the model, the soft hinge between axes *z*_4_ and *z*_5_ is stretched during folding, and its width is denoted as *d*. Figure 1i shows the model predictions on the input-output curves of dihedral angles in the thick-panel origami (input dihedral angle: ${{\varphi }}_{1}$, output dihedral angles: ${{\varphi }}_{2},{{\varphi }}_{3},{{\varphi }}_{4}$) are identical with that of the corresponding zero-thickness origami (input dihedral angle: ${{\varphi }}_{1}^{o}$, output dihedral angles: ${{\varphi }}_{2}^{o},{{\varphi }}_{3}^{o},{{\varphi }}_{4}^{o}$) during the folding process as long as the width *d* satisfies the relation:

1 $${d}{=}\frac{2{a}\,\sin {{{{{\rm{\beta }}}}}}\,\cos {{{{{{\rm{\varphi }}}}}}}_{5}}{\sin {{{{{\rm{\alpha }}}}}}},$$

where *α* ∈ [0, π], *β* ∈ [0, π], *α* + *β* = π, and *φ*_5_ ∈ [0, π/2]. Detailed derivation process of this equation is presented in Supplementary Note 6. This indicates that the kinematics of the thick-panel origami is consistent with that of the corresponding zero-thickness origami, and the thick-panel origami is foldable. To further demonstrate this excellent foldability, as shown in Fig. 1j, we print a nonzero thickness plane-symmetric four-creases single vertex origami (diameter: 120 mm, thickness: 6 mm). The origami panel can be fully folded by stretching the soft hinge to accommodate the gap between the thick panels. It should be noted that the folding of a nonzero thickness four-crease single vertex origami may also be achieved using strained joints. However, the strained joint method requires special design and processing steps to make the stiff material accommodate large deformations during origami folding^43^. In addition, compared with the highly stretchable soft hinges in this work, the low fatigue resistance of the strained joints may be a concern^26^.

### Self-locking origami unit: from push to pull

**Fig. 2:**
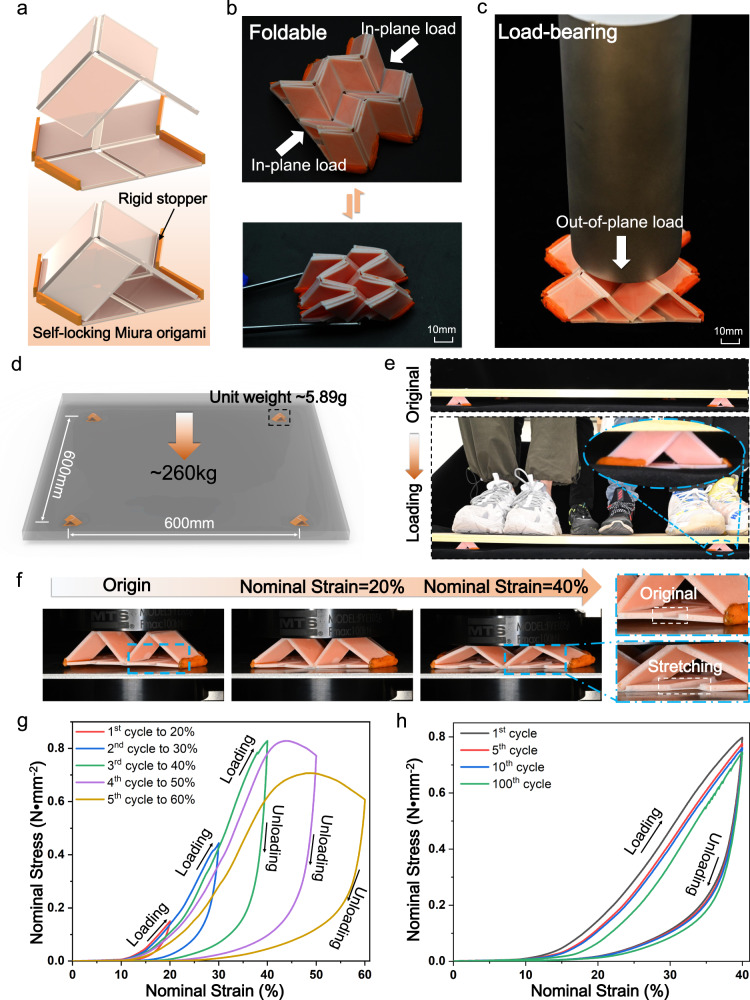
Design and mechanical performance of self-locking thick-panel origami. **a** Assembly of 3D printed flat thick-panel Miura-origami sheets to achieve self-locking property. **b** 3D printed self-locking thick-panel origami structure with 2 × 2 units can be folded along with in-plane direction. **c** The origami structure can support a heavy out-of-plane load. **d**, **e** Demonstrates that thick-panel origami units can withstand a load over 11000 times of its own weight without failure. **f** Snapshots of a 3D printed self-locking thick-panel origami structure with 2 × 2 units under compression. **g** Gradient loading-unloading results of the 3D printed self-locking thick-panel origami structure with 2 × 2 units. **h** Cyclic compression results of the 3D printed self-locking thick-panel origami structure with 2 × 2 units under 40% compressive strain.

A generic degree-4 vertex origami (including Miura-origami) exhibits continuous motion that transform itself from a flat state to a fully folded one^44^. This folding can be stopped when locking mechanism is applied to constrain the origami’s boundary. Here, we assemble two 3D printed flat thick-panel Miura-origami panels to form a 3D self-locking origami unit^14^. As shown in Fig. 2a, the self-locking origami unit is formed by stacking the partially folded upper origami sheet on the flat bottom origami sheet. The upper and bottom origami panels have the same projection area. To constrain the upper partially folded origami sheet from recovering to its original flat configuration, we print two rigid stoppers at the ends of the bottom origami sheet. Following this strategy, as shown in Fig. 2b, we design and assembly a 3D self-locking thick-panel origami structure with 2 × 2 units. Since we use soft glue to attach the upper origami panels to the bottom ones, we can easily fold the origami structure by applying an in-plane load (Supplementary Movie 1). More importantly, as demonstrated in Fig. 2c, the self-locking mechanism makes the origami structure capable of supporting a heavy out-of-plane load. To further demonstrate this excellent load capacity, in Fig. 2d, we place four self-locking thick-panel origami units in the corners of a wood plate. The distance between each origami unit is 600 mm. In Fig. 2e, we added a heavy load to the four origami units via letting four male adults (weighting over 260 kg) stand on the wood plate. Remarkably, the four origami units can withstand a load of over 260 kg which means each origami unit could support more than 65 kg that is over 11000 times of its weight (5.89 g). Different from the conventional self-locking origami structures with local buckling behavior^17,45^, the 3D printed self-locking thick-panel origami structure exhibits a unique “push-to-pull (P2P)” deformation mode (Fig. 2f), which transfers the out-of-plane compressive deformation to the in-plane stretch of bottom TPU hinges. As shown in Fig. 2g, when the compressive strain is less than 40%, the 3D printed self-locking origami structure with P2P deformation mode exhibits excellent elasticity. The highest load that a thick-panel origami structure with 2 × 2 units could support is 3073 N (0.82 MPa × 3747.56 mm^2^ in Fig. 2g). More importantly, the P2P deformation mode imparts great mechanical cyclability to the self-locking origami structure. As presented in Fig. 2h, it could sustain 100 cycles of 40% compressive strain without obvious failure (Supplementary Note 7 and Supplementary Movie 2).

**Fig. 3:**
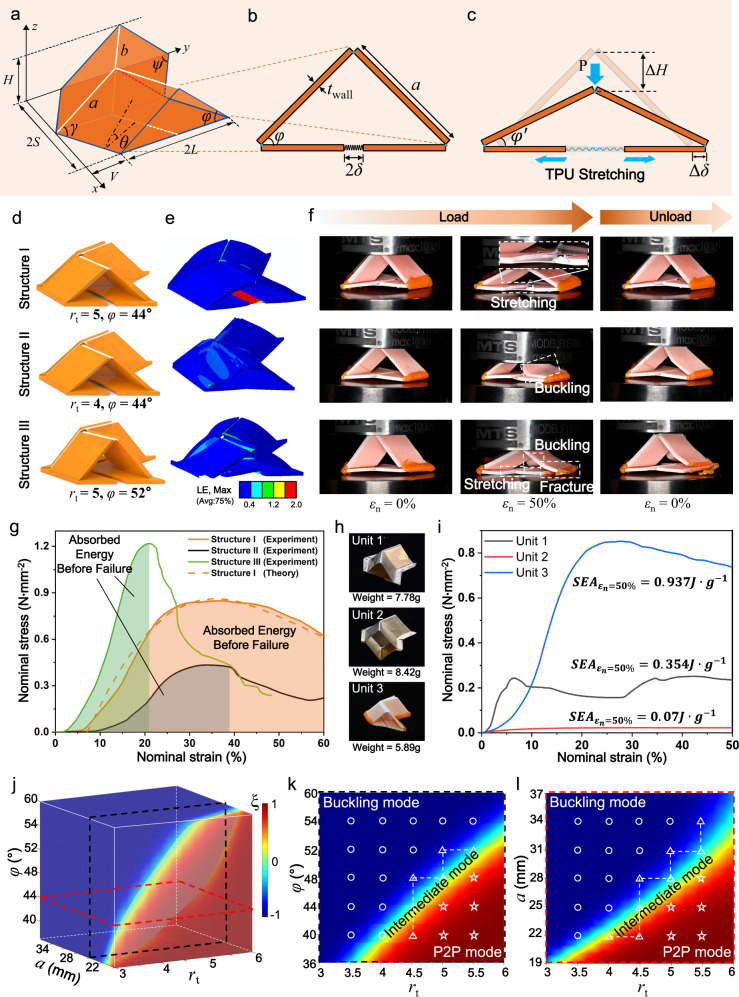
Deformation mechanism of 3D printed self-locking thick-panel origami units. **a** Geometric model of a self-locking origami unit. **b** The section view of self-locking thick-panel origami unit. **c** Illustration of the self-locking thick-panel origami unit under out-of-plane load with P2P deformation mode. **d** Self-locking thick-panel origami units with different geometric parameters. **e** FEA simulation results of three self-locking thick-panel origami units. (The color scale represents the logarithmic strain (LE) of the material ranging from 0 to 2.) **f** Snapshots of loading and unloading process of the three origami units under 50% compressive strain. **g** Nominal stress–strain curves of three origami structures. The filled area represents the absorbed energy before failure. **h** Snapshots of three origami unit with different materials and configurations. **i** Compressive tests to compare the SEA of the three origami units. **j** 3D design space map that distinguish the geometric parameter spaces from P2P, to intermediate or buckling deformation mode. Cut view of 3D design space map with **k** *a* = 25 mm, and **l** *φ* = 44°. (Circles, triangles and stars represent the self-locking thick-panel origami unit with buckling mode, intermediate mode and P2P mode, respectively.).

To have in-depth understanding on the deformation mechanism of the 3D printed self-locking thick-panel origami unit, we develop a theoretical model to mathematically describe its deformation behavior and guide the structure design. In Fig. 3a, we present the zero-thickness model of the self-locking origami unit where the upper part consists of four identical parallelograms with sides *a* and *b* as well as acute angle *γ*. The dihedral folding angle between the upper parallelograms and the bottom *xy* plane is *θ* (*θ* ∈ [0, π/2]). The angle between the upper and bottom origami panels is *φ*. The outer dimensions of the self-locking origami unit can be given by

2 $${{S}}={{b}}\cdot \,\sin \psi,\,{{L}}={{a}}\cdot \,\cos \varphi,\,{{H}}={{{{{\rm{a}}}}}}\cdot \,\sin \gamma \cdot \,\sin \theta .$$

Additional useful relationships are sin*γ* ‧ sin*θ* = sin*φ*, tan*ψ* = cos*θ* ‧ tan*γ*, where *ψ* ∈ [0, *γ*], and *φ* ∈ [0, *γ*]. Derivation details for Eq. (2) can be found in Supplementary Note 8. Figure 3b presents the cross-section of the self-locking thick-panel origami unit where the wall thickness of origami unit is *t*_wall_, the length and the thickness of the soft hinge in the bottom origami sheet soft hinge is 2*δ* and *t*_TPU_, respectively. Figure 3c illustrates the P2P deformation mode of the self-locking thick-panel origami unit when it is under compression. When a vertically force *P* is applied, the height of the self-locking thick-panel origami unit decreases by Δ*H*, and the upper panels rotates by *φ*–*φ*’. The origami structure can transfer the vertically applied compressive force *P* from the upper panels to the horizontal stretch of the soft hinge that connects the bottom panels. The soft hinge is stretched by Δ*δ*/*δ* where 2*δ* and 2Δ*δ* are the original length and the extension of the soft hinge. Comparing the side profile of the origami structure before and after compression, we have the geometric relation:

3 $$a(\cos {{{{{\rm{\varphi }}}}}}{\prime} -\,\cos {{{{{\rm{\varphi }}}}}})=\varDelta \delta,$$

which allows us to use *φ* and Δ*δ* to calculate *φ*’. The stress–strain behavior of the soft hinge can be captured by Mooney–Rivlin model with strain energy density function as $W={C}_{10}({I}_{1}-3)+{C}_{01}({I}_{2}-3)+{D}_{1}^{-1}{(J-1)}^{2}$ where ${I}_{1}$, ${I}_{2}$, ${I}_{3}$ (${I}_{3}=J$) are the three invariants, and *C*_10_, *C*_01_, and *D*_1_ are the material parameters. Based on the force analysis of the origami structure and detailed derivations on the stress–strain behavior of the soft hinge (Supplementary Note 8 and S9), we have the relation between force *P* and the half-extension of the soft hinge Δ*δ*:

4 $$P=\frac{8b\cdot {t}_{{{{{{\rm{TPU}}}}}}}}{\cot {{\varphi }}^{\prime} -\mu }\bigg\{{C}_{10}\left[\left(1+\frac{\varDelta \delta }{\delta }\right)-{\left(1+\frac{\varDelta \delta }{\delta }\right)}^{-2}\right]+{C}_{01}\left[1-{\left(1+\frac{\varDelta \delta }{\delta }\right)}^{-3}\right]\bigg\},$$

where *μ* is the friction coefficient between the bottom panel and the ground that supports the origami structure.

As shown in Supplementary Fig. 10, we attribute the vertical displacement Δ*H* of the origami structure’s top ridge into two parts:

5 $$\varDelta H=\varDelta {H}_{1}+\varDelta {H}_{2},$$

where $\varDelta {H}_{1}$ is resulted from the geometric change, and $\varDelta {H}_{2}$ is resulted from the axial compression of the upper panels. Based on Supplementary Note 10,

6 $$\varDelta {H}_{1}=a(\sin {{\varphi }}-\,\sin {{\varphi }}^{\prime} ),\,{{{{{\rm{and}}}}}}\,\varDelta {H}_{2}=P\cdot a\cdot {(4\sin {{\varphi }}\cdot \sin {{\varphi }}^{\prime} \cdot {E}_{{{{{{\rm{panel}}}}}}}\cdot b\cdot {t}_{{{{{{\rm{wall}}}}}}})}^{-1},$$

where *E*_panel_ is Young’s modulus of the upper panel. By combing Eqs. (3)–(6), we can find that Δ*H* is a function of *P*:

7 $$\varDelta H=\varDelta H(P)=\varDelta {H}_{1}(P)+\varDelta {H}_{2}(P).$$

The upper panels start to buckle when the applied force *P* is greater than the critical buckling load *P*_cr_ which can be calculated as

8 $${P}_{{{{{{\rm{cr}}}}}}}=4b\cdot {t}_{{{{{{\rm{wall}}}}}}}\cdot {{\sigma }}_{{{{{{\rm{cr}}}}}}}\cdot \,\sin {{\varphi }}^{\prime},$$

where ${\sigma }_{{{{{{\rm{cr}}}}}}}$ is the critical buckling stress which can be calculated following the buckling theory for thin plates^46^ (Supplementary Note 11).

In order to ensure that the self-locking thick-panel origami structure deforms by following the P2P deformation mode to achieve better mechanical performance, it is critical to find an appropriate combination of design parameters. In Fig. 3d, to demonstrate this significance, we present three self-locking thick-panel origami structures that have different wall thickness of the upper sheet (*t*_wall_) and the angle between the upper and lower panels (*φ*) while keeping the other parameters the same (detailed geometric parameters of these three structures were shown in Supplementary Table 3). Structure I with *a* = 25 mm, thickness ratio *r*_t_ = 5 (*r*_t_ = *t*_wall_/*t*_TPU,_ *t*_TPU_ = 0.4 mm), and *φ* = 44° deforms by following the P2P deformation mode. In Fig. 3e, the finite element analysis (FEA) shows that the soft hinge of Structure I is stretched by 200%. Details on the FEA modeling can be found in Supplementary Note 12. In contrast, by reducing *r*_t_ from 5 to 4, Structure II fails due to buckling of the upper panel; by increasing *φ* from 44° to 52°, Structure III fails due to buckling of the upper panel as well as facture of the corner between the upper and bottom panels. Figure 3f presents the experimental snapshots of the three structures which exhibit the same deformation modes as FEA simulations predicted. Remarkably, Structure I could fully recover to its undeformed configuration after unloading due to the elastic deformation on the soft hinges, while Structure II and III fails the full recovery due to the damages on the panels.

Figure 3g presents the “stress–strain” behaviors of the three samples where the nominal stress ${\sigma }_{n}$ is defined as *P*/(4*SL*), and the nominal strain ${\varepsilon }_{n}$ is defined as Δ*H*/*H*. Structure I performs a nearly linear stress–strain behavior when the nominal stress is less than 0.8 N · mm^−2^. After that, the structure further deforms without obvious stress variation until the nominal strain is greater than 50%. This ductile-like stress–strain behavior makes the Structure I able to absorb higher mechanical energy before failure (5536 mJ) which can be calculated as ${U}={\int }_{{V}}{\int }_{0}^{{\varepsilon }_{f}}{\sigma }_{n}{{{{{\rm{d}}}}}}{\varepsilon }_{n}d\Omega$, where *V* is the volume of the origami unit, and *ε*_f_ is the failure strain. In comparison, Structure II has lower stiffness and exhibits a sudden failure when the nominal stress is greater than 0.4 N · mm^−2^ due to buckling of the upper rigid panel; Structure III has a higher stiffness than Structure I, but it absorbs much lower mechanical energy (2157 mJ) due to the sudden fracture when the nominal strain is greater than 20%. Moreover, as shown in Supplementary Note 13, the FEA simulations reveal that the soft hinges in Structure I store more than 50% mechanical energy due to the large deformation. In contrast, in Structure II or III, more mechanical energy is stored in the upper panel due to buckling. Figure 3g also shows that the theoretical prediction of the stress–strain curve of Structure I agrees well with the experimental result, which indicates the theoretical model can successfully capture the stress–strain curve of self-locking thick-panel origami structure, and be further used to guide its design. In addition, as shown in Fig. 3h, we also fabricated origami unit based on previous works^17^. Unit 1 and Unit 2 were made of brass (fabrication process can be found in Supplementary Note 14), and have the same key parameters (*a* = 25 mm, *φ* = 44°) as the origami unit cell exhibiting P2P deformation mode (Unit 3). The masses of the three samples are similar. Figure 3i presents the compression results of the three samples, where Unit 3 has the highest specific absorption energy at 50% strain (*SEA*_*ε*=50%_ = 0.93 J · g^−1^). Besides, as shown in Supplementary Fig. 15, Unit 3 was able to recover to its initial undeformed state even after it was compressed by 50%, while the other two samples underwent plastic deformation and could not fully recover.

We determine whether the self-locking thick-panel origami structure exhibits P2P or buckling deformation mode by comparing the magnitude of critical buckling force *P*_cr_ and ideal maximum force *P*_max_ of the structure. *P*_cr_ can be calculated from Eq. (8). *P*_max_ can be determined when ${{{{{\rm{d}}}}}}P/{{{{{\rm{d}}}}}}(\varDelta H)$ = 0, and the relation between *P* and Δ*H* can be found from Eq. (7). If the origami structure exhibits P2P deformation mode, the different between *P*_cr_ and *P*_max_ (Δ*P* = *P*_cr_ − *P*_max_) is positive. If the origami structure exhibits buckling mode, Δ*P* is negative. In order to quantify and visualize whether the self-locking thick-panel structure exhibits P2P or buckling deformation mode, we modify Sigmoid function as *ξ* = *A* · [1 + exp(−*B* · Δ*P*)]^−1^ + *C* where we set *A* = 2 and *C* = −1 to make the return value *ξ* in the range of [−1, 1], and *B* is a fitting parameter. Figure 3j presents the return value *ξ* in the 3D design space including geometric parameters of the length of upper panel *a*, the thickness ratio *r*_t_ (*r*_t_ = *t*_wall_/*t*_TPU_), and the angle between upper and bottom panels *φ*. To visually distinguish P2P and buckling in the 3D design space, we set *B* = 10 so that the red space or blue space indicate P2P or buckling deformation mode, respectively. In addition, when *P*_cr_ is near *P*_max_, or Δ*P* is close to zero, there exists the intermediate mode in which the soft hinge (TPU) is first stretched under compression, and then the upper panels buckle as the compression increases further (such as Structure III in Fig. 3d–f and Supplementary Fig. 16). In Fig. 3k, the intermediate mode can be found in the rainbow color region (−0.4 < *ξ* < 0.4) of the 3D design space. Figure 3k, l presents the cut views of Fig. 3j by setting *a* = 25 mm or *φ* = 44°, respectively. To validate the theoretical model prediction, we conducted compression experiments on the self-locking origami units with different design parameters (hollow circles, triangles, and stars) which confirm that all the deformation modes observed from experiments agree well with theoretical results. Representative experiments can be found in Supplementary Fig. 16. Moreover, this theoretical model could also be used to guide us to improve compressive strength of the self-locking thick-panel origami units by tuning the combination of *r*_t_ and *φ*, or *a* and *r*_t_ (Supplementary Fig. 17).

### Programmable mechanical response of 3D printed multi-unit self-locking thick-panel origami

**Fig. 4:**
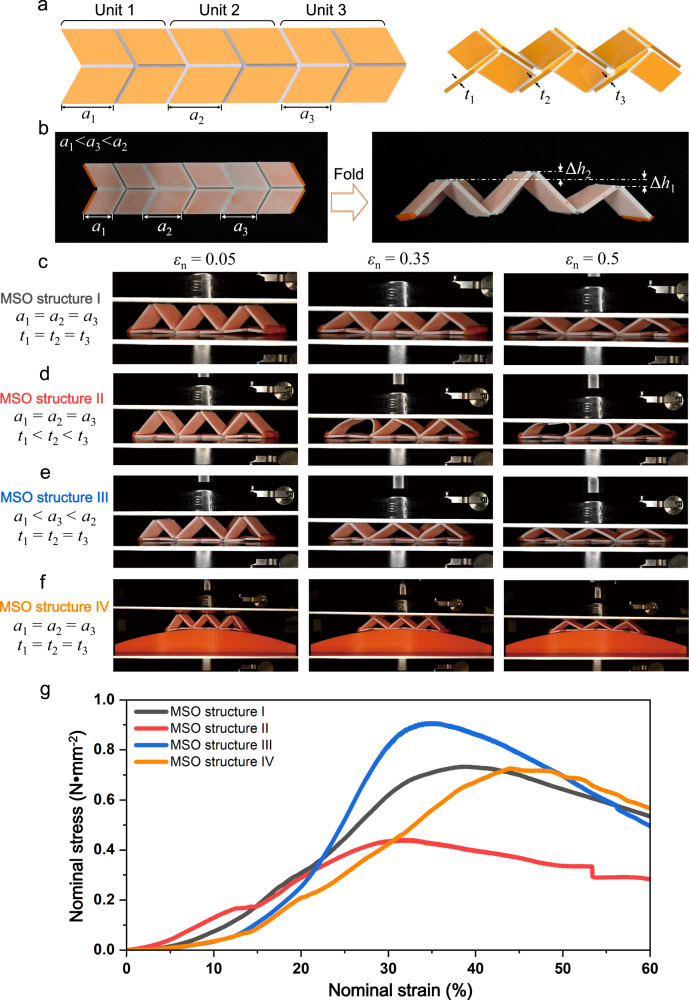
Programmable mechanical response of MSO structures. **a** Geometric parameters of MSO structure. **b** The demonstration shows that origami structures with different heights can be achieved by adjusting the wall length of each unit. **c**–**f** Snapshots of MSO structure I-IV under compression. **g** Nominal stress–strain behaviors of four different MSO structures.

To further demonstrate the high flexibility on design and manufacturing of the 3D printed self-locking thick-panel origami structures, we fabricate multi-unit self-locking thick-panel origami (MSO) with various design parameters. Figure 4a illustrates that the upper origami pattern consists of three Miura-origami units where the wall length (*a*_1_, *a*_2_, *a*_3_) and the wall thickness (*t*_1_, *t*_2_, *t*_3_) of each origami unit is tunable. As demonstrated in Fig. 4b, we can achieve an origami structure where the heights of the three peaks are different by assigning the wall length of each unit to following the order as *a*_1_ < *a*_3_ < *a*_2_. Figure 4c–f presents the compressions of MSO structures with different design parameters. Detailed design parameters are listed in Table 1.

**Table 1:** Geometry design parameters of MSO structures

Structure	Base	*a*_1_ (mm)	*a*_2_ (mm)	*a*_3_ (mm)	*t*_1_ (mm)	*t*_2_ (mm)	*t*_3_ (mm)
I	Flat	25	25	25	2.2	2.2	2.2
II	Flat	25	25	25	1.4	1.8	2.2
III	Flat	22	28	25	2.2	2.2	2.2
IV	Curved	25	25	25	2.2	2.2	2.2

Figure 4c and Supplementary Movie 3 show the compression of the MSO structure I consisting of three identical Miura-origami units (*a* = 25 mm, *t* = 2.2 mm) which follow the P2P deformation mode. In Fig. 4d and Supplementary Movie 4, MSO structure II has the same wall length as MSO structure I (*a* = 25 mm), but its wall thickness varies 1.4 to 2.2 mm. When MSO structure II is under compression, Units 1 and 2 buckle sequentially due to the thin wall thickness, but Unit 3 follows the P2P mode. Compared with MSO structure I, MSO structure III has the same wall thickness (*t* = 2.2 mm), but different wall lengths (*a*_1_ = 22 mm, *a*_2_ = 28 mm, *a*_3_ = 25 mm). As shown in Fig. 4e and Supplementary Movie 5, under compression, Unit 2 deforms first as it is highest; then, Unit 3 and Unit 1 are compressed sequentially. Since the three units have sufficient wall thickness, they all deform by following the P2P deformation mode. In addition, the printed MSO structure also exhibits excellent adaptability to curved surface. As demonstrated in Fig. 4f and Supplementary Movie 6, MSO structure IV has the same geometric parameters as MSO structure I, but is placed on a curved surface. Its great flexibility makes MSO structure IV well conform to the curved surface without losing its load capacity as each unit deforms by following the P2P deformation mode. The high design flexibility and excellent adaptability to curved surface of the MSO structures open up a range of application possibilities. As shown in Supplementary Fig. 18, the MSO can be used to form the energy absorbing layer for safety helmets.

Figure 4g presents the nominal stress–strain curves of the four MSO structures. MSO structure I exhibits the typical nominal stress–strain curve of the thick-panel origami unit following the P2P deformation mode. Compared with MSO structure I, MSO structure II has lower strength as its Unit 1 and Unit 2 buckle under compression. At the very beginning of compression, MSO structure III has lower stiffness as only Unit 2 is compressed. When the compression proceeds, the stiffness of MSO structure III increases gradually as the Unit 3 and Unit 1 are compressed sequentially. MSO structure III yields at 0.9 MPa which is the highest among the four MSO structures. Although MSO structures I and IV have the same geometric parameters, the initial stiffness of MSO structure IV is lower as it is placed on the curve surface and only Unit 2 is compressed at beginning. The further compression makes MSO structure IV yield at 0.73 MPa which is about the same as that of MSO structure I when the three identical units undergo the P2P deformation mode.

### Impact of 3D printed self-locking thick-panel origami-based mechanical metamaterials

**Fig. 5:**
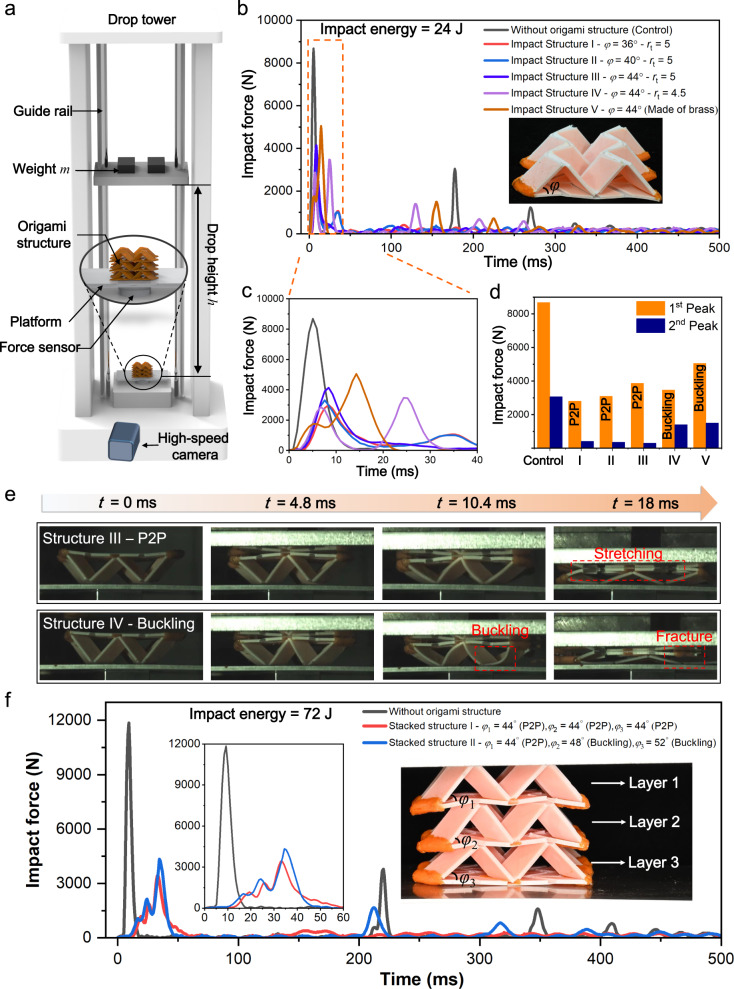
Impact absorption of 3D printed self-locking thick-panel origami-based mechanical metamaterials. **a** Illustration of a self-built drop tower for impact test. **b** Impact response of structures under impact energy of 24 J. **c** Impact response of structures under impact energy of 24 J within the first 40 ms. **d** 1^st^ and 2^nd^ peak impact force for Impact Structures with impact energies of 24 J. **e** Snapshots of the impact process of Impact Structure III and IV. **f** Impact responses of stacked origami metamaterials under impact energy of 72 J.

The P2P deformation mode also endow the self-locking thick-panel origami with excellent capability of absorbing impact energy. As illustrated in Fig. 5a, we investigate impact energy absorption of the self-locking thick-panel origami structures on a self-built drop tower (Supplementary Fig. 19) where the impact is generated by dropping weight *m* from height *h*, and origami structure is placed on the platform below which a force sensor is attached. A high-speed camera is set in front of the origami structure to capture its deformation during impact. The impact energy can be adjusted by tuning *m* and *h*, and calculated as *E* = *mgh*.

Figure 5b presents the impact force variation over time, and compares the impact energy absorption capability of four origami structures with different geometric parameters. In addition, we also conducted the drop test without placing any origami structure on the platform. There are two key indicators to evaluate a structure’s capability of absorbing impact energy^47^: (i) the value of the first peak impact force; (ii) whether there is a secondary impact. As shown in Fig. 5b, during the drop test (*E* = 24 J, *m* = 8.25 kg, *h* = 0.3 m) without origami structure (control test), the first peak impact force appears at 5.04 ms with magnitude of 8641.76 N and span of 12.74 ms. We also observe the second and third peaks of impact force with magnitude of 3026.91 N and 1249.36 N due to the rebounding of the weight and the re-collision between weight and platform. After placing the structure with P2P deformation mode (Impact Structure I–III) on the platform, as shown in Fig. 5c, we could observe only one peak of impact force with lower magnitude (3000~4000 N) and broader span (~33 ms). It should be noted that the collision between the weight and Impact Structure I–III is inelastic, and no obvious secondary peak of impact force could be found since the two parts stick together after the first collision. Remarkably, when Structure I (*φ* = 36°) is placed, the first peak impact force is reduced by 65.5% from 8641.76 N to 2981.4 N.

Different from Impact Structure I–III, Impact Structure IV exhibits buckling deformation mode which leads to the elastic collision between the weight and Impact Structure IV. As shown in Fig. 5b, the second to the fourth peaks of impact force can be clearly found at 25.02 ms, 129.69 ms, 207.40 ms which are caused by the rebounding of the weight and re-collision between the weight and Impact Structure IV. Moreover, tuning the geometric parameters allows us to adjust the origami structure’s impact absorption capability. Figure 5e presents the snapshots of the impact tests applied to Impact Structures III and IV, the impact processes of Impact Structures III and IV were shown in Supplementary Movie 7 and S8, respectively. On Impact Structure III, the P2P deformation mode transfers the vertical impact force to the large stretch of TPU, while the deformations on the rigid panels are relatively small. In comparison, the impact force results in buckling on the rigid panels of Impact Structure IV which eventually leads to the fracture of them.

In addition, we also conducted drop tests on the brass-made 2 × 2 Miura origami structures (Impact Structure V in Fig. 5b) made of brass (*a* = 25 mm, *φ* = 44°, *t*_brass_ = 0.2 mm). The first peak impact force is 5078.7 N which is much higher than that of Impact Structure I–III, and a secondary peak impact force of 1518.54 N can be found at 155 ms. Details about the drop tests on the brass-made Miura origami structures can be found in Supplementary Note 20. As shown in Fig. 5d, the five self-locking origami structures were subjected to the same impact energy (24 J), and the Impact Structures with P2P deformation mode had a lower first-peak impact force and almost negligible second-peak impact force. However, Impact Structures with buckling deformation mode has not only a higher first-peak impact force but also a significant second-peak impact force. Comparing the drop testing results of the five structures, we conclude that the self-locking thick-panel origami structures with P2P deformation mode exhibit better impact energy absorption performance than brass-based origami structure with similar geometry and weight.

We can further improve the impact energy absorption by stacking multiple layers of the self-locking thick-panel origami structures in vertical direction. As shown in Fig. 5f, a three-layer origami structure is able to absorb the impact energy of 72 J (*m* = 12.85 kg, *h* = 0.57 m) and could reduce the peak impact force by 72.1% from 11897.41 N to 3314.37 N. Again, the P2P deformation mode imparts better capability of absorbing impact energy. When the multi-layer origami structure consists of three origami units which all follow P2P deformation mode (*φ*_1_ = *φ*_2_ = *φ*_3_ = 44°), the collision between the weight and origami structure is inelastic, and no further peak of impact force is observed. In comparison, when there are two origami units exhibiting buckling deformation mode (*φ*_2_ = 48° and *φ*_3_ = 52°), the collision between the weight and the multi-layer origami structure becomes elastic, and multiple peaks of impact force are observed due to re-collisions between the two parts.

In summary, we report a universal manufacturing and design approach for thick-panel origami structures that are fabricated via FDM multimaterial 3D printing. We propose the wrapping strategy that provides the hinge with robust interfacial between rigid and soft parts so that the printed origami hinge can fold more than 100 times and be stretched by 300% without failure. Through stacking two pieces of printed thick-panel origami panels into a predetermined configuration, we developed 3D self-locking Miura-origami structures that are foldable and capable of withstanding more than 100 compression cycles with a 40% compression strain through unconventional push-pull (P2P) deformation modes. In addition, we build a theoretical model to mathematically describe deformation behavior of the self-locking thick-panel origami and guide its structure design. To demonstrate the high flexibility on its design and manufacturing, we fabricate multi-unit self-locking thick-panel origami (MSO) with various design parameters which exhibits high mechanical tunability and adaptability to the loading substrates with various geometries. Finally, we demonstrate the P2P deformation mode endows the 3D self-locking thick-panel origami with superior capability to absorb impact energy, which can greatly reduce the peak impact force as well as avoid secondary impact even under high impact energy (for example, a three-layer self-locking origami structure can reduce the peak impact force from 11897.41 N to 3314.37 N under a 72 J impact). This manufacturing and design strategy for thick-panel foldable origami metamaterials here paves a new way towards practical engineering applications.

## Methods

### Multimaterial 3D printing of thick-panel origami

PLA (Polylactic acid), TPU (Thermoplastic polyurethanes), ABS (Acrylonitrile butadiene styrene) and CFRP (Carbon fiber reinforced polymer) filaments for printing were used as received from Polymaker (China).

The thick-panel origami 3D model consists of two parts, one part is given TPU material, the other part is given PLA material, in which PLA part is completely wrapped and connected by TPU part, and the thickness for the wrapping and connecting is set to 0.4 mm (*t*_TPU_). The two 3D parts were designed and exported to STL files via Solidworks (V2016, Dassault Systèmes Simulia Corp., USA). After that, these two STL files were firstly assembled in Ultimaker Cura software and then were sliced with layer thickness set as 0.2 mm. The printing temperature for PLA material and TPU material was 210 °C and 220 °C, respectively. The bed temperature during the printing was set to be 60 °C. The printing speed for PLA material and TPU material were set to 60 mm·min^−1^ and 30 mm·min^−1^, respectively.

### Uniaxial tensile experiments

The size of RSR specimens used for tensile experiments were all 70 mm × 15 mm × 1 mm. The hinge length and thickness of the RSR specimen were 3 mm and 0.4 mm, respectively. Tensile tests were performed using an MTS machine (10 kN load cell, USA) at room temperature with a loading speed of 2 mm·min^−1^.

### Cyclic compression test

Cyclic compression test of 3D printed a thick-panel Miura-origami sheet with 2 × 2 units was performed using an MTS machine (10 kN load cell, USA) at room temperature. The thickness of rigid panels and soft hinges were set to 2.2 mm and 0.4 mm. The loading and unloading speeds were both set to 10 mm·min^−1^. Detailed experiment process and result can be found in Supplementary Note 1.

Cyclic compression test of a 3D printed self-locking thick-panel origami structure was performed using an MTS machine (10 kN load cell, USA) at room temperature. The TPU thickness (*t*_TPU_), soft hinge length (2*δ)*, unit wall width (*b*), and acute angle (*γ*) of 3D printed self-locking thick-panel origami structures mentioned in this test were 0.4 mm, 3.5 mm, 21.6 mm and 60.9°, respectively. The loading and unloading speeds were both set to 2 mm·min^−1^. Detailed experiment process and result can be found in Supplementary Note 6.

### Quasi-static compression tests

Uniaxial compression test of 3D printed self-locking thick-panel origami structures were performed using an MTS machine (10 kN load cell, USA) at room temperature with a crosshead speed of 2 mm·min^−1^.

### Finite element analysis (FEA)

To predict the deformation on the self-locking thick-panel origami unit, FEA simulations were conducted by using the commercially available software package ABAQUS (V6.14, Dassault Systèmes Simulia Corp., USA). We use the hyperelastic Mooney–Rivlin model with strain energy density function $W={C}_{10}({I}_{1}-3)+{C}_{01}({I}_{2}-3)+\frac{1}{{D}_{1}}{(J-1)}^{2}$ to describe the nonlinear material behavior of TPU. The material coefficients were set as *C*_10_ = 1.57 MPa, *C*_01_ = 2.18 MPa, which were obtained by fitting the uniaxial tensile experiments of TPU. The rigid panel formed by TPU wrapped PLA was simplified to a composite material and the modulus of the composite material was set to be 369.5 MPa by curve fitting based on experiment results. Tie constraints were used to connect the rigid panels to the soft hinges. Detailed FEA verification results were presented in Supplementary Note 12. The 3D model of self-locking thick-panel origami units (Structure I, Structure II, Structure III) were constructed and analyzed on ABAQUS/Explict (Simulia, Dassault Systemes). Solid tetrahedron quadratic element (element type C3D10M) was used to mesh the structures. The displacement was applied to the rigid plate above the self-locking thick-panel structure to simulate the quasi-static compression.

## Supplementary information

Supplementary Information

Description of Additional Supplementary Files

Supplementary Movie 1

Supplementary Movie 2

Supplementary Movie 3

Supplementary Movie 4

Supplementary Movie 5

Supplementary Movie 6

Supplementary Movie 7

Supplementary Movie 8

## Data Availability

The data generated in this study are provided in manuscript and Supplementary Information.
