# Supplementary material for: Multimaterial 3D printed self-locking thick-panel origami metamaterials
Source: Nat Commun. 2023 Mar 23;14:1607. doi: 10.1038/s41467-023-37343-w (PMC10036479; doi:10.1038/s41467-023-37343-w)
Supplement: Supplementary file 1 — Supplementary Information [file 41467_2023_37343_MOESM1_ESM.pdf]

Supplementary Information for

Multimaterial 3D Printed Self-locking Thick-panel Origami Metamaterials

Haitao Ye[1,2,3], Qingjiang Liu[1,2], Jianxiang Cheng[1,2], Honggeng Li[1,2], Bingcong Jian[1,2], Rong Wang[1,2], Zechu Sun[1,2], Yang Lu[3,4,5]* and Qi Ge[1,2]*

[1]Shenzhen Key Laboratory of Soft Mechanics & Smart Manufacturing, Southern University of Science and Technology, Shenzhen, 518055, China
[2]Department of Mechanical and Energy Engineering, Southern University of Science and Technology, Shenzhen 518055, China
[3]Department of Mechanical Engineering, City University of Hong Kong, Kowloon, Hong Kong SAR, China
[4]Nano-Manufacturing Laboratory (NML), Shenzhen Research Institute of City University of Hong Kong, Shenzhen, China
[5]Department of Mechanical Engineering, The University of Hong Kong, Pokfulam Road, Hong Kong SAR, China
*Corresponding author: Qi Ge, e-mail: geq@sustech.edu.cn; Yang Lu, e-mail: ylu1@hku.hk

This PDF file includes:

Supplementary Text
Supplementary Figures 1 to 20
Supplementary Tables 1 to 3

Supplementary Notes

Supplementary Note 1. Cyclic test on a thick-panel Miura-origami sheet with 2 × 2 units.

To evaluate the fatigue resistance of the printed thick-panel Miura-origami during the folding-unfolding process, we conduct a cyclic compression test using MTS machine (100 N load cell, USA). The thick-panel Miura-origami sheet with 2 × 2 units is placed on the platform in a pre-folded shape (Supplementary Figure 1c). We set this position as initial compression position and loading speed as 10 mm·min^{-1}. As shown in Supplementary Figure 1b, the maximum compressive displacement is 45 mm (65% compressive strain) when the origami structure is flat-folded. During the cyclic test, the peak force in each cyclic is stable and no obvious force drops are observed (as shown in Supplementary Figure 1a). The printed Miura-origami sheet with 2 × 2 units can sustain more than 100 cycles compression without obvious failure.

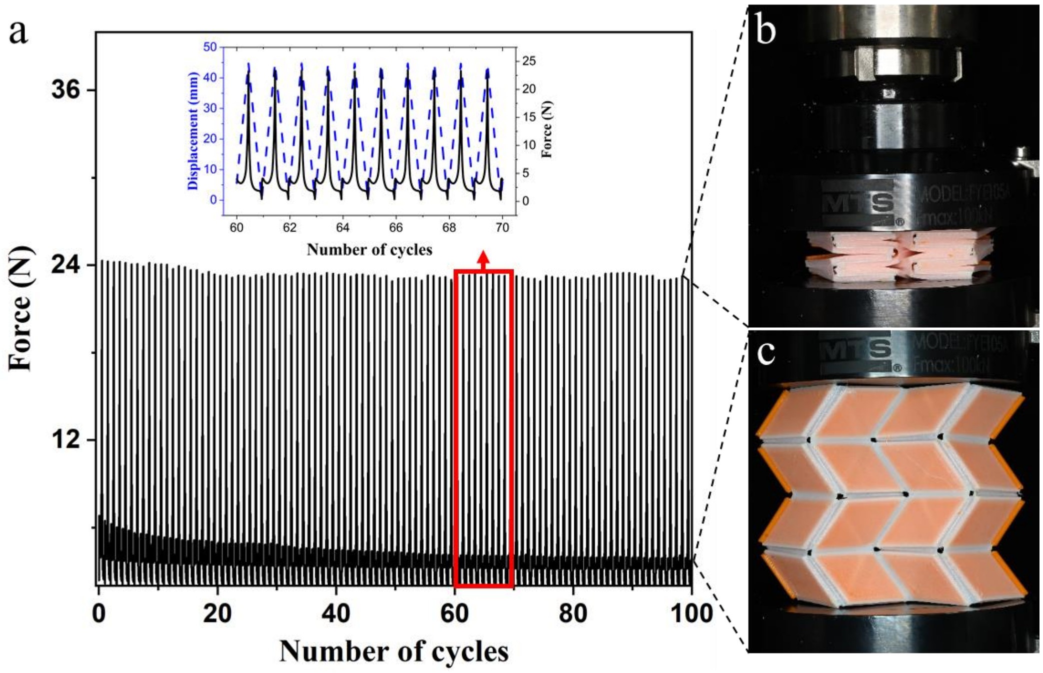

Supplementary Figure 1. Cyclic compression test for a printed thick-panel Miura-origami sheet with 2 × 2 units. (a) Force versus number of cycles during compression. (b) Snapshot of the origami sheet at fully folded shape. (c) Snapshot of the origami sheet at unfolded shape.

Supplementary Note 2. Poor interfacial bonding of an origami sheet where rigid PLA panels directly printed onto the soft TPU substrate.

To demonstrate the poor interfacial bonding of origami structure printed by the conventional method, as shown in Supplementary Figure 2a, we printed a Miura-origami sheet with 2 × 2 units where the rigid PLA panels were directly printed onto the soft TPU substrate. Due to the poor interfacial bonding, obvious delamination between PLA and TPU can be clearly found by simply bending the Miura-origami sheet (Supplementary Figure 2b).

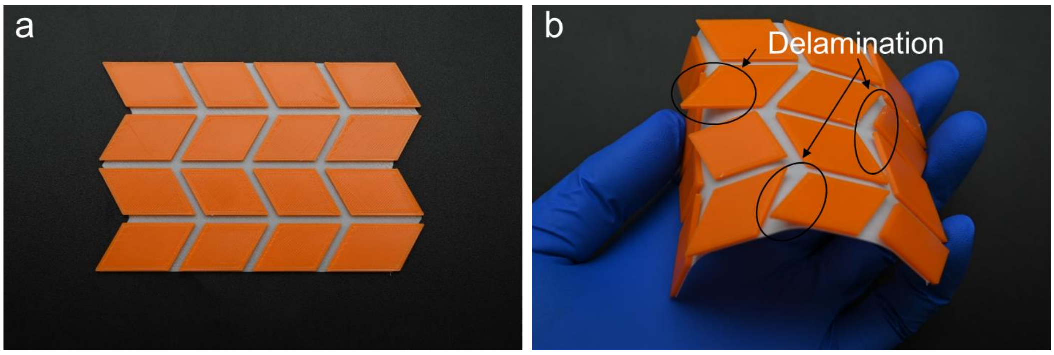

Supplementary Figure 2. Demonstration of Poor interfacial bonding of an origami sheet printed by the conventional method. (a) A Miura-origami sheet with 2 × 2 units where the rigid PLA panels were directly printed onto the soft TPU substrate. (b) Delamination between PLA and TPU can be easily found by bending the Miura-origami sheet.

Supplementary Note 3. Effects on printing parameters (Raster angle and layer thickness) on printed multimaterial structures.

We performed three tensile tests with different raster angles (0°, 45°, 90°), and the tensile results are shown in Supplementary Figure 3a. The specimen with raster angle of 0°(the raster angle we used in this work) has excellent tensile properties and higher

tensile strength compared to the other two specimens. Of the three specimens, the specimen with a raster angle of 45 °was the first to fail at the strain of 231%. In addition, we also explored the effect of the printed layer thickness (0.1, 0.15, 0.2 mm) on the mechanical properties of the structure, and the results are shown in Supplementary Figure 3b, where we can find that the effect of the printed layer thickness on its mechanical properties is almost negligible.

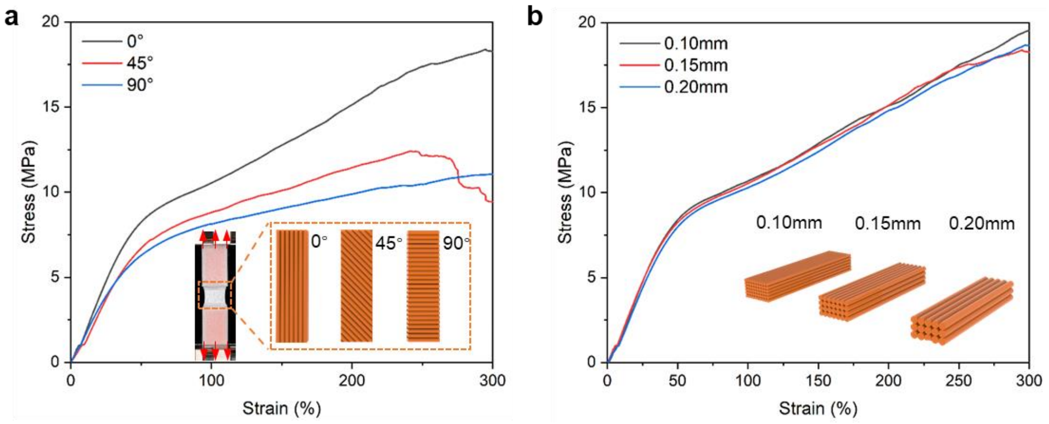

Supplementary Figure 3. Tensile results of specimens with different printing parameters. (a) Stress-strain curves of the printed specimen with different raster angles by wrapping-based method. (b) Stress-strain behavior of the printed specimen with different layer thicknesses by wrapping-based method.

Supplementary Note 4. Tensile results of pure TPU, rigid-soft-rigid (RSR) hinges with PLA, ABS, and CFRP.

The proposed rigid-soft coupled multimaterial 3D printing strategy is not material-dependent, and applicable to couple various rigid and soft materials. To demonstrate this advantage, we printed three RSR hinges where PLA, acrylonitrile butadiene styrene (ABS), and carbon fiber reinforced polymer (CFRP) were used to form the main parts of rigid panels. Supplementary Figure 4 compares the stress-strain curves of the RSR

hinges with that of the pure TPU sample. Three RSR hinges exhibit similar stress-strain behavior with that of pure TPU sample, and can stretch to over 300% without failure, which proves the universality of the multimaterial 3D printing strategy based on this work.

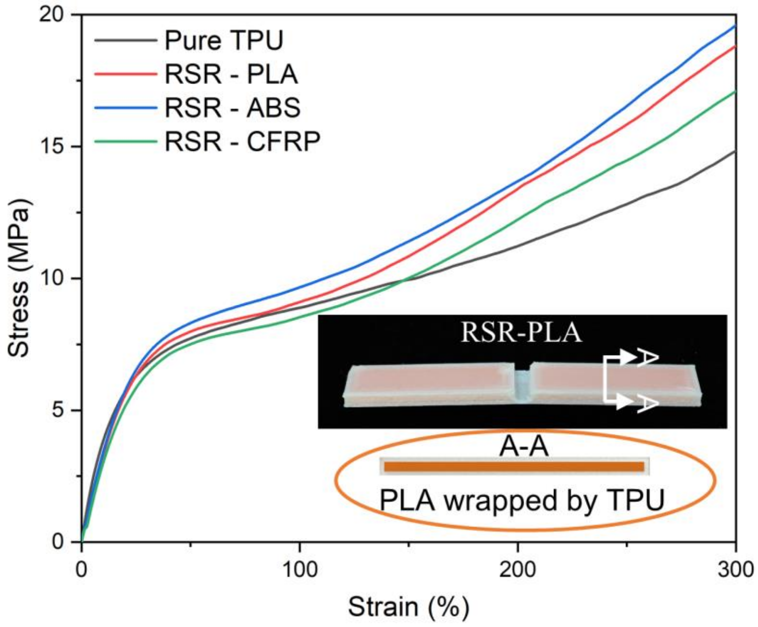

Supplementary Figure 4. Comparison on the stress-strain behavior between the pure TPU and RSR hinges where the rigid parts are mainly made of PLA, ABS and CFRP respectively. Inset: snapshot of a RSR-PLA hinge with the rigid parts where the PLA is wrapped by TPU.

Supplementary Note 5. Tensile performance of RSR samples fabricated by conventional and wrapping-based methods.

We fabricated two rigid-soft-stiff (RSR) samples by conventional method and wrapping-based method, respectively. The stress-strain curve of the sample printed by the wrapping-based method shows significant advantages in both fracture strain and strength (Supplementary Figure 5).

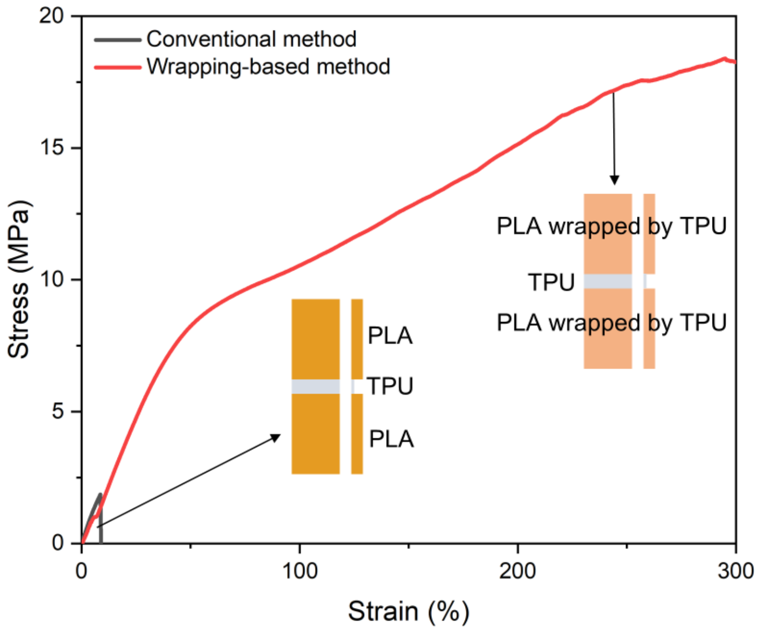

Supplementary Figure 5. Stress-strain behavior of the RSR hinges made through conventional and wrapping-based method.

Supplementary Note 6. Kinematic modeling of 4-creases single vertex thick-panel origami.

In this work, we proposed the soft hinge shift technique (Figure 1d) for thick-panel origami, which can only be realized in the origami structure formed by stretchable soft hinges. To better popularize this technique in various thick plate origami applications of origami patterns with four-crease single-vertex, we further modified the kinematic model based on the previous work published by Chen et al. [1,2], and the foldability of multimaterial 3D printed thick-panel origami is proved by the following derivation.

In this four-crease single-vertex origami model, the kinematic analysis of the proposed 4-creases single vertex thick-panel origami is carried out based on D-H convention (Supplementary Figure 6a). The linkage mechanism consists of a series of joints and links, and the links connected to each joint can be defined as a joint coordinate system.

The transformation from one joint coordinate system to the adjacent next joint coordinate system can obtain a transformation matrix. By analogy, until the last joint coordinate system is transformed, the transformation of all joint coordinate systems is combined, and finally the overall transformation matrix of the linkage mechanism can be obtained. The transformation of the whole process is regarded as rotation and translation of the coordinate system.

In this plane-symmetric four-crease single vertex thick-panel origami model (Figure 1h), both sides of the upper soft hinge (Z_4 and Z_5) are set as parallel axes and the distance between axes is d which is changeable due to the highly stretchable TPU. Z_1, Z_2 and Z_3 are set as rotation joints.

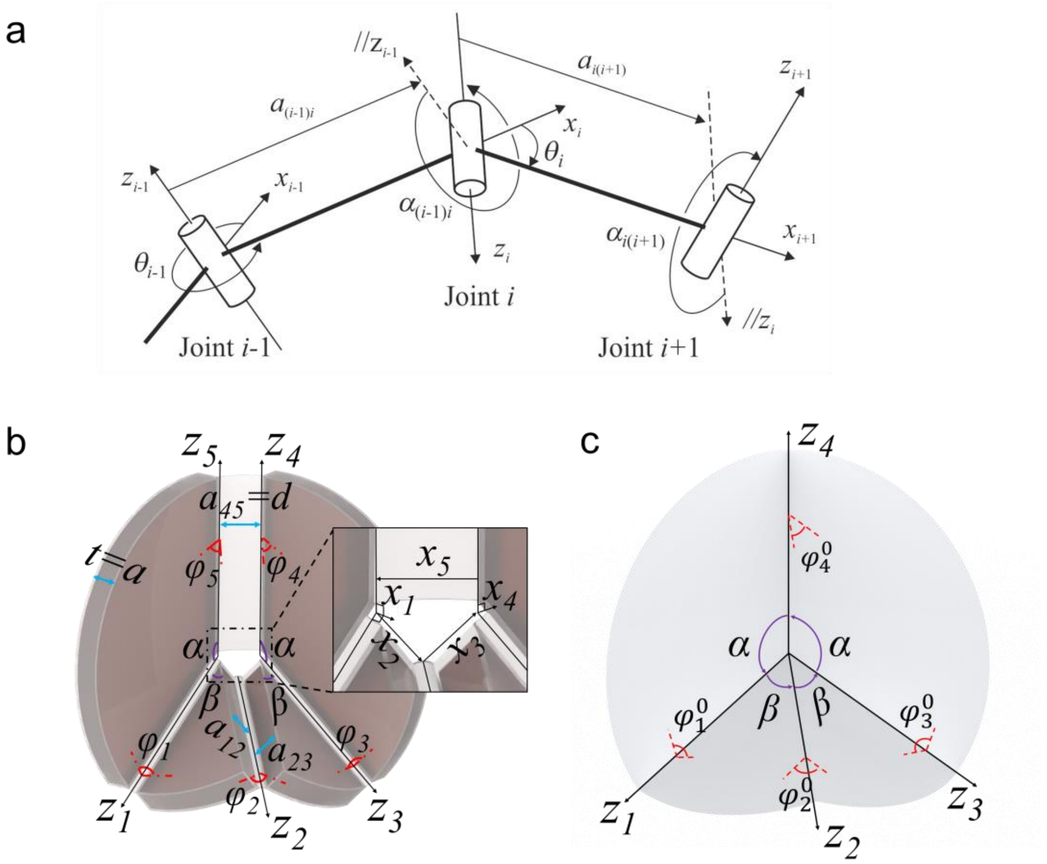

Supplementary Figure 6. Kinematic modeling of plane-symmetric 4-creases single vertex thick-panel origami. (a) Illustration of D-H convention. Diagram of four-crease single vertex thick-panel origami model (b), and zero-thickness origami model (c).

There is a series of spatial links where adjacent rigid links are connected only by revolute joints, forming a loop. Such linkages can be analyzed using the matrix method with DH notations introduced by Denavit and Hartenberg. The coordinate system of each joint is established as follows:

(1) If axis Z_i is rotating joint, Z_i coincides with joint axis and the rotation direction is determined according to the right-hand rule. If axis Z_i is a sliding joint, Z_i coincides with the direction of the joint's linear motion.

(2) Axis X_i is defined as the common vertical line between the joint axis Z_{i-1} and the joint axis Z_i. If the axes of two joints are parallel, there are countless common vertical lines between them. If the axes of two adjacent joints intersect, the common vertical distance between them is zero. At this time, the axis is defined as a straight line perpendicular to the plane formed by the two axes.

(3) The origin O_i is defined as the intersection of the axis X_i and the axis Z_i.

(4) The length of the connecting rod a_i is defined as the length of the common vertical line from the axis Z_i to the axis Z_{i+1}, and the direction along X_{i+1} the axis is positive.

(5) The rotation angle $\alpha_{i(i+1)}$ is defined as the angle from the axis Z_i to the axis Z_{i+1}. Whether the rotation is positive or negative is judged by right hand rule. It is positive along the X_{i+1} axis.

(6) According to the right-hand rule, the positive rotation around the axis X_i is

positive.

The joint offset R_i is defined as the distance from the axis X_i to the common vertical line of the axis X_{i+1}, and the direction along the axis Z_i is positive.

(7) The joint rotation angle θ_i is defined as the angle from the axis X_i to the axis X_{i+1}, and the rotation around the axis Z_i is positive according to the right-hand rule.

Through the above steps, the relative relationship between the two joints *i* and *i*+1 is established. The transformation formula and its expanded matrix are:

$$\mathbf{T}_{(i+1)i} = \mathbf{Rot}(z,\theta_i)\bullet\mathbf{Trans}(0,0,R_i)\bullet\mathbf{Trans}(a_{i(i+1)},0,0)\bullet\mathbf{Rot}(x,\alpha_{i(i+1)})$$
$$=\begin{bmatrix}\cos\theta_i & -\sin\theta_i & 0 & 0\\ \sin\theta_i & \cos\theta_i & 0 & 0\\ 0 & 0 & 1 & 0\\ 0 & 0 & 0 & 1\end{bmatrix}\begin{bmatrix}1 & 0 & 0 & 0\\ 0 & 1 & 0 & 0\\ 0 & 0 & 1 & R_i\\ 0 & 0 & 0 & 1\end{bmatrix}\begin{bmatrix}1 & 0 & 0 & a_{i(i+1)}\\ 0 & 1 & 0 & 0\\ 0 & 0 & 1 & 0\\ 0 & 0 & 0 & 1\end{bmatrix}\begin{bmatrix}1 & 0 & 0 & 0\\ 0 & \cos\alpha_{i(i+1)} & -\sin\alpha_{i(i+1)} & 0\\ 0 & \sin\alpha_{i(i+1)} & \cos\alpha_{i(i+1)} & 0\\ 0 & 0 & 0 & 1\end{bmatrix} \tag{1}$$

$$\mathbf{T}_{(i+1)i} = \begin{bmatrix}\cos\theta_i & -\cos\alpha_{i(i+1)}\sin\theta_i & \sin\alpha_{i(i+1)}\sin\theta_i & a_{i(i+1)}\cos\theta_i\\ \sin\theta_i & \cos\alpha_{i(i+1)}\cos\theta_i & -\sin\alpha_{i(i+1)}\cos\theta_i & a_{i(i+1)}\sin\theta_i\\ 0 & \sin\alpha_{i(i+1)} & \cos\alpha_{i(i+1)} & R_i\\ 0 & 0 & 0 & 1\end{bmatrix} \tag{2}$$

$$\mathbf{T}^{-1}_{(i+1)i} = \mathbf{T}_{i(i+1)} = \begin{bmatrix}\cos\theta_i & \sin\theta_i & 0 & -a_{i(i+1)}\\ -\cos\alpha_{i(i+1)}\sin\theta_i & \cos\alpha_{i(i+1)}\cos\theta_i & \sin\alpha_{i(i+1)} & -R_i\sin\alpha_i\\ \sin\alpha_{i(i+1)}\sin\theta_i & -\sin\alpha_{i(i+1)}\cos\theta_i & \cos\alpha_{i(i+1)} & -R_i\cos\alpha_i\\ 0 & 0 & 0 & 1\end{bmatrix} \tag{3}$$

$$\mathbf{T}_{21}\mathbf{T}_{32}\ldots\mathbf{T}_{i(i-1)}\mathbf{T}_{i1} = \mathbf{I} \tag{4}$$

$$\mathbf{T}_{21}\mathbf{T}_{32}\mathbf{T}_{43}\mathbf{T}_{54}\mathbf{T}_{15} = \mathbf{I} \tag{5}$$

The four-crease single vertex thick-panel origami model (Supplementary Figure 6b) has the following relationship:

$$\alpha_{12} = -\alpha_{23} = -\beta,\ \alpha_{34} = -\alpha_{51} = -\alpha,\ \alpha_{45} = 0, \quad (6)$$

$$a_{12} = a_{23} = a,\ a_{34} = a_{51} = 0,\ a_{45} = d. \quad (7)$$

According to the convention, the plus and minus signs here indicate its direction.

To use a more intuitive representation of joint rotation angle θ_i, we use φ_i to express the relationships of two dihedral of panels. The mathematical notations for the transformations are expressed as

$$\varphi_1 = 2\pi - \theta_1,\ \varphi_2 = \theta_2,\ \varphi_3 = 2\pi - \theta_3,\ \varphi_4 = \pi - \theta_4,\ \varphi_5 = \pi - \theta_5. \quad (8)$$

The physical meaning of φ_i is depicted in Figure 1h.

And there is no offset between x_i and x_{i+1} along the axis Z_i. We can obtain

$$R = [R_1, R_2, R_3, R_4, R_5] = [0, 0, 0, 0, 0]. \quad (9)$$

Equation (5) can be rewritten as $\mathbf{T}_{32}\mathbf{T}_{43} = \mathbf{T}_{21}^{-1}\mathbf{T}_{15}^{-1}\mathbf{T}_{54}^{-1}$, the equality of elements (3, 3) in both sides gives:

$$(\mathbf{T}_{32}\mathbf{T}_{43})_{3,3} = (\mathbf{T}_{21}^{-1}\mathbf{T}_{15}^{-1}\mathbf{T}_{54}^{-1})_{3,3}, \quad (10)$$

$$\begin{aligned}&\cos\alpha_{23}\cos\alpha_{34} - \sin\alpha_{34}\cos\theta_3\sin\alpha_{23} - \cos\alpha_{45}(\cos\alpha_{12}\cos\alpha_{51} - \sin\alpha_{12}\cos\theta_1\sin\alpha_{51})\\ &-\sin\alpha_{45}\left(-\cos\alpha_{51}\cos\theta_5\sin\alpha_{12}\cos\theta_1 - \cos\alpha_{12}\sin\alpha_{51}\cos\theta_5 + \sin\alpha_{12}\sin\theta_1\sin\theta_5\right) = 0.\end{aligned} \quad (11)$$

After the specific parameters are brought in, equation can be simplified as

$$\cos\beta\cos(-\alpha) - \sin(-\alpha)\sin\beta\cos\theta_3 - \left(\cos\beta\cos\alpha - \sin(-\alpha)\sin\beta\cos\theta_1\right) = 0, \quad (12)$$

Because $\theta_1 \in (\pi, 2\pi)$, $\theta_2 \in (\pi, 2\pi)$, equation (12) can be simplified to $\cos\theta_1 = \cos\theta_3$, it's easy to obtain

$$\theta_1 = \theta_3. \quad (13)$$

And after substituting θ_1 and θ_3 with $\theta_1 = 2\pi - \varphi_1$ and $\theta_3 = 2\pi - \varphi_1$

respectively, equation (13) can be transformed to

$$\varphi_1 = \varphi_3 . \tag{14}$$

Thus, the first conclusion is obtained.

The equality of elements (3, 3) can also be expressed as

$$\left(\mathbf{T}_{43}\mathbf{T}_{54}\mathbf{T}_{15}\right)_{3,3} = \left(\mathbf{T}_{32}^{-1}\mathbf{T}_{21}^{-1}\right)_{3,3}, \tag{15}$$

$$\begin{aligned}&-\cos\alpha_{12}\cos\alpha_{23} + \sin\alpha_{23}\cos\theta_2\sin\alpha_{12} + \cos\alpha_{51}\left(\cos\alpha_{34}\cos\alpha_{45} - \sin\alpha_{45}\cos\theta_4\sin\alpha_{34}\right)\\ &-\sin\alpha_{51}\cos\theta_5\left(\cos\alpha_{45}\cos\theta_4\sin\alpha_{34} + \cos\alpha_{34}\sin\alpha_{45}\right) + \sin\alpha_{51}\sin\theta_5\sin\alpha_{34}\sin\theta_4 = 0.\end{aligned} \tag{16}$$

After the specific parameters are brought in, equation can be simplified as

$$-\cos^2\beta - \sin^2\beta\cos\theta_2 + \cos^2\alpha + \sin^2\alpha\cos\theta_5\cos\theta_4 - \sin^2\alpha\sin\theta_4\sin\theta_5 = 0, \tag{17}$$

$$-\cos^2\beta + \cos^2\alpha + \sin^2\beta\cos\theta_2 - \sin^2\alpha\cos\left(\theta_4 + \theta_5\right) = 0 , \tag{18}$$

$$\begin{aligned}\cos\left(\theta_4 + \theta_5\right) &= \frac{-\cos^2\beta + \cos^2\alpha + \sin^2\beta\cos\theta_2}{\sin^2\alpha} = \frac{-\cos^2\beta + 1 - \sin^2\alpha + \sin^2\beta\cos\theta_2}{\sin^2\alpha}\\ &= \frac{\sin^2\beta - \sin^2\alpha + \sin^2\beta\cos\theta_2}{\sin^2\alpha} = \frac{\sin^2\beta\left(1+\cos\theta_2\right) - \sin^2\alpha}{\sin^2\alpha} = \frac{\sin^2\beta}{\sin^2\alpha}\left(1+\cos\theta_2\right) - 1.\end{aligned} \tag{19}$$

After substituting θ_4 and θ_5 with $\theta_4 = \pi - \varphi_4$ and $\theta_5 = \pi - \varphi_5$ respectively, we can obtain

$$\cos\left(2\pi - \varphi_4 - \varphi_5\right) = \frac{\sin^2\beta}{\sin^2\alpha}\left(1+\cos\left(\varphi_2\right)\right) - 1. \tag{20}$$

This can be simplified to the second conclusion as

$$\cos\left(\varphi_4 + \varphi_5\right) = 1 + \frac{\sin^2\beta}{\sin^2\alpha}\left(\cos\varphi_2 - 1\right). \tag{21a}$$

Due to symmetry， we obtain $\varphi_4 = \varphi_5$. Hence, equation (21) can be rewritten as

$$\cos(2\varphi_4) = 1 + \frac{\sin^2\beta}{\sin^2\alpha}(\cos\varphi_2 - 1) \tag{21b}$$

Because $\mathbf{T}_{15}\mathbf{T}_{21}\mathbf{T}_{32}\mathbf{T}_{43}\mathbf{T}_{54} = \mathbf{I}$, the equality of elements (3, 3) can be expressed as

$$\left(\mathbf{T}_{15}\mathbf{T}_{21}\mathbf{T}_{32}\right)_{3,3} = \left(\mathbf{T}_{54}^{-1}\mathbf{T}_{43}^{-1}\right)_{3,3}, \tag{22}$$

$$\begin{aligned}&-\cos\alpha_{34}\cos\alpha_{45} + \sin\alpha_{45}\cos\theta_4\sin\alpha_{34}\\ &-\sin\alpha_{23}\cos\theta_2\left(\cos\alpha_{51}\sin\alpha_{12} + \cos\alpha_{12}\cos\theta_1\sin\alpha_{51}\right)\\ &+\cos\alpha_{23}\left(\cos\alpha_{12}\cos\alpha_{51} - \sin\alpha_{12}\cos\theta_1\sin\alpha_{51}\right) + \sin\alpha_{23}\sin\theta_2\sin\alpha_{51}\sin\theta_1 = 0,\end{aligned} \tag{23}$$

$$\begin{aligned}&-\cos\alpha + 0 - \sin\beta\cos\theta_2\left(\cos\alpha\sin(-\beta) + \cos(-\beta)\sin\alpha\cos\theta_1\right)\\ &+\cos\beta\left(\cos\alpha\cos(-\beta) - \sin(-\beta)\sin\alpha\cos\theta_1\right) + \sin\beta\sin\alpha\sin\theta_2\sin\theta_1 = 0,\end{aligned} \tag{24}$$

$$\begin{aligned}&-\cos\alpha + \sin^2\beta\cos\alpha\cos\theta_2 + \cos^2\beta\cos\alpha - \sin\beta\cos\beta\sin\alpha\cos\theta_1\cos\theta_2\\ &+\sin\beta\cos\beta\sin\alpha\cos\theta_1 + \sin\beta\sin\alpha\sin\theta_2\sin\theta_1 = 0,\end{aligned} \tag{25}$$

$$\cos\alpha\sin^2\beta\left(\cos\theta_2 - 1\right) + \sin\beta\cos\beta\sin\alpha\cos\theta_1\left(1 - \cos\theta_2\right) + \sin\beta\sin\alpha\sin\theta_2\sin\theta_1 = 0, \tag{26}$$

$$\left(\cos\alpha\sin^2\beta - \sin\beta\cos\beta\sin\alpha\cos\theta_1\right)\left(1 - \cos\theta_2\right) = \sin\beta\sin\alpha\sin\theta_2\sin\theta_1, \tag{27}$$

$$\frac{1-\cos\theta_2}{\sin\theta_2} = \frac{\sin\alpha\sin\theta_1}{\cos\alpha\sin\beta - \cos\beta\sin\alpha\cos\theta_1}. \tag{28}$$

Because $\dfrac{1-\cos\theta_2}{\sin\theta_2} = \tan\dfrac{\theta_2}{2}$, we can simplify (S28) to

$$\tan\frac{\theta_2}{2} = \frac{\sin\alpha\sin\theta_1}{\cos\alpha\sin\beta - \cos\beta\sin\alpha\cos\theta_1}. \tag{29}$$

Because of

$$\varphi_1 = 2\pi - \theta_1, \varphi_2 = \theta_2, \tag{30}$$

the third conclusion to express the relationship between φ_1 and φ_2 can be obtained as

$$\tan\frac{\varphi_2}{2} = \frac{\sin\alpha\sin\varphi_1}{\cos\beta\sin\alpha\cos\varphi_1 - \cos\alpha\sin\beta}. \tag{31}$$

After the transformation of $\mathbf{T}_{21}\mathbf{T}_{32}\mathbf{T}_{43}\mathbf{T}_{54}\mathbf{T}_{15}=\mathbf{I}$, the equality of elements (3,3) can be obtained as followed:

$$\left(\mathbf{T}_{21}\mathbf{T}_{32}\mathbf{T}_{43}\right)_{3,3}=\left(\mathbf{T}_{15}^{-1}\mathbf{T}_{54}^{-1}\right)_{3,3}, \tag{32}$$

$$d\sin\alpha_{51}\sin\theta_5+a\sin\theta_2\sin\alpha_1=0, \tag{33}$$

Based on equation (33), we can obtain the relationship between *d* and φ_5. The fourth conclusion is

$$d=\frac{-a\sin(-\beta)\sin\theta_2}{\sin\alpha\sin\theta_5}=\frac{a\sin\beta\sin\varphi_2}{\sin\alpha\sin(\pi-\varphi_5)}=\frac{2a\sin\beta\sin\varphi_5\cos\varphi_5}{\sin\alpha\sin\varphi_5}=\frac{2a\sin\beta\cos\varphi_5}{\sin\alpha}. \tag{34}$$

The single vertex zero-thickness rigid origami can be modelled as a spherical linkage (Supplementary Figure 6c). For a spherical linkage, the distances between adjacent links are zero because the axes of revolute joints meet at a point, and thus the transformation matrix $\mathbf{T}_{(i+1)i}$ can be simplified to $Q_{(i+1)i}$, where

$$Q_{(i+1)i}=\begin{bmatrix}\cos\theta_i & -\cos\alpha_{i(i+1)}\sin\theta_i & \sin\alpha_{i(i+1)}\sin\theta_i\\ \sin\theta_i & \cos\alpha_{i(i+1)}\cos\theta_i & -\sin\alpha_{i(i+1)}\cos\theta_i\\ 0 & \sin\alpha_{i(i+1)} & \cos\alpha_{i(i+1)}\end{bmatrix}. \tag{35}$$

The four-crease single vertex zero-thickness origami model has the following relationship:

$$\alpha_{12}^0=\alpha_{23}^0=\beta,\ \alpha_{34}^0=\alpha_{41}^0=\alpha, \tag{36}$$

$$\varphi_1^0=\pi-\theta_1^0,\ \varphi_2^0=\theta_2^0-\pi,\ \varphi_3^0=\pi-\theta_3^0,\ 2\varphi_4^0=\pi-\theta_4^0. \tag{37}$$

After transformation, we can have:

$$\theta_1^0=\pi-\varphi_1^0,\ \theta_2^0=\pi+\varphi_2^0,\ \theta_3^0=\pi-\varphi_3^0,\ \theta_4^0=\pi-2\varphi_4^0. \tag{38}$$

After the transformation of $\mathbf{Q}_{21}\mathbf{Q}_{32}\mathbf{Q}_{43}\mathbf{Q}_{14}=\mathbf{I}$, the equality of elements (3,3) can be obtained as followed:

$$\left(\mathbf{Q}_{32}\mathbf{Q}_{43}\right)_{3,3}=\left(\mathbf{Q}_{21}^{-1}\mathbf{Q}_{14}^{-1}\right)_{3,3}, \tag{39}$$

$$\begin{aligned}&\cos\alpha_{23}^{0}\cos\alpha_{34}^{0}-\cos\alpha_{12}^{0}\cos\alpha_{41}^{0}-\sin\alpha_{34}^{0}\left(-\cos\varphi_{3}^{0}\right)\sin\alpha_{23}^{0}\\&+\sin\alpha_{12}^{0}\left(-\cos\varphi_{1}^{0}\right)\sin\alpha_{41}^{0}=0,\end{aligned} \tag{40}$$

$$\cos\beta\cos\alpha-\cos\beta\cos\alpha+\sin\alpha\cos\varphi_{3}^{0}\sin\beta-\sin\beta\cos\varphi_{1}^{0}\sin\alpha=0, \tag{41}$$

$$\cos\varphi_{1}^{0}=\cos\varphi_{3}^{0}. \tag{42}$$

Combine the actual physical meaning of the equation, we can obtain the fifth conclusion

$$\varphi_{1}^{0}=\varphi_{3}^{0}, \tag{43}$$

which is same to first conclusion shown in equation (14).

After the transformation of $\mathbf{Q}_{21}\mathbf{Q}_{32}\mathbf{Q}_{43}\mathbf{Q}_{14}=\mathbf{I}$, the equality of elements (3,3) can be obtained as followed:

$$\left(\mathbf{Q}_{21}\mathbf{Q}_{32}\right)_{3,3}=\left(\mathbf{Q}_{14}^{-1}\mathbf{Q}_{43}^{-1}\right)_{3,3}, \tag{44}$$

$$\begin{aligned}&\cos\alpha_{12}^{0}\cos\alpha_{23}^{0}-\cos\alpha_{34}^{0}\cos\alpha_{41}^{0}-\sin\alpha_{23}^{0}(-\cos\varphi_{2}^{0})\sin\alpha_{12}^{0}\\&+\sin\alpha_{41}^{0}[-\cos(2\varphi_{4}^{0})]\sin\alpha_{34}^{0}=0,\end{aligned} \tag{45}$$

$$\cos^{2}\beta-\cos\alpha^{2}+\sin^{2}\beta\cos\varphi_{2}^{0}-\sin^{2}\alpha\cos(2\varphi_{4}^{0})=0, \tag{46}$$

$$\cos(2\varphi_{4}^{0})=\frac{\cos^{2}\beta-\cos\alpha^{2}}{\sin^{2}\alpha}+\frac{\sin^{2}\beta}{\sin^{2}\alpha}\cos\varphi_{2}^{0}, \tag{47}$$

$$\cos(2\varphi_{4}^{0})=\frac{1+\cos^{2}\beta-1-\cos\alpha^{2}}{\sin^{2}\alpha}+\frac{\sin^{2}\beta}{\sin^{2}\alpha}\cos\varphi_{2}^{0}, \tag{48}$$

$$\cos(2\varphi_{4}^{0})=\frac{\sin^{2}\alpha-\sin^{2}\beta}{\sin^{2}\alpha}+\frac{\sin^{2}\beta}{\sin^{2}\alpha}\cos\varphi_{2}^{0}. \tag{49}$$

We can obtain the sixth conclusion

$$\cos(2\varphi_{4}^{0})=1+\frac{\sin^{2}\beta}{\sin^{2}\alpha}(\cos\varphi_{2}^{0}-1). \tag{50}$$

This equation is same to the second conclusion shown in equation (21b).

After the transformation of $\mathbf{Q}_{21}\mathbf{Q}_{32}\mathbf{Q}_{43}\mathbf{Q}_{14}=\mathbf{I}$, the equality of elements (3,3) can be obtained as followed:

$$\left(\mathbf{Q}_{21}\mathbf{Q}_{32}\mathbf{Q}_{43}^{-1}\right)_{3,3}=\left(\mathbf{Q}_{14}^{-1}\right)_{3,3}, \tag{51}$$

$$\begin{aligned}&-\cos\alpha_{41}^{0}+\cos\alpha_{34}^{0}[\cos\alpha_{12}^{0}\cos\alpha_{23}^{0}-\sin\alpha_{23}^{0}(-\cos\varphi_{2}^{0})\sin\alpha_{12}^{0}]\\&-\sin\alpha_{34}^{0}(-\cos\varphi_{3}^{0})[\cos\alpha_{23}^{0}(-\cos\varphi_{2}^{0})\sin\alpha_{12}^{0}+\cos\alpha_{12}^{0}\sin\alpha_{23}^{0}]\\&+\sin\alpha_{34}^{0}\sin\varphi_{3}^{0}\sin\alpha_{12}^{0}\sin(-\varphi_{2}^{0})=0,\end{aligned} \tag{52}$$

$$\begin{aligned}&-\cos\alpha+\cos\alpha(\cos^{2}\beta+\sin^{2}\beta\cos\varphi_{2}^{0})\\&+\sin\alpha\cos\varphi_{3}^{0}(-\cos\beta\cos\varphi_{2}^{0}\sin\beta+\cos\beta\sin\beta)\\&-\sin\alpha\sin\varphi_{3}^{0}\sin\beta\sin\varphi_{2}^{0}=0,\end{aligned} \tag{53}$$

$$\begin{aligned}&\cos\alpha(\cos^{2}\beta+\sin^{2}\beta\cos\varphi_{2}^{0}-1)\\&+\sin\alpha\cos\varphi_{3}^{0}\cos\beta\sin\beta(1-\cos\varphi_{2}^{0})\\&=\sin\alpha\sin\varphi_{3}^{0}\sin\beta\sin\varphi_{2}^{0},\end{aligned} \tag{54}$$

$$\begin{aligned}&\cos\alpha\sin^{2}\beta(\cos\varphi_{2}^{0}-1)\\&+\sin\alpha\cos\varphi_{3}^{0}\cos\beta\sin\beta(1-\cos\varphi_{2}^{0})\\&=\sin\alpha\sin\varphi_{3}^{0}\sin\beta\sin\varphi_{2}^{0},\end{aligned} \tag{55}$$

$$(\sin\alpha\cos\varphi_{3}^{0}\cos\beta-\cos\alpha\sin\beta)(1-\cos\varphi_{2}^{0})=\sin\alpha\sin\varphi_{3}^{0}\sin\varphi_{2}^{0}, \tag{56}$$

$$\frac{\sin\alpha\cos\varphi_{3}^{0}\cos\beta-\cos\alpha\sin\beta}{\sin\alpha\sin\varphi_{3}^{0}}=\frac{\sin\varphi_{2}^{0}}{1-\cos\varphi_{2}^{0}}, \tag{57}$$

$$\cot\frac{\varphi_{2}^{0}}{2}=\frac{\sin\alpha\cos\varphi_{3}^{0}\cos\beta-\cos\alpha\sin\beta}{\sin\alpha\sin\varphi_{3}^{0}}, \tag{58}$$

$$\tan\frac{\varphi_{2}^{0}}{2}=\frac{\sin\alpha\sin\varphi_{3}^{0}}{\sin\alpha\cos\beta\cos\varphi_{3}^{0}-\cos\alpha\sin\beta}. \tag{59}$$

Because $\varphi_{1}^{0}=\varphi_{3}^{0}$, we can obtain the seventh conclusion

$$\tan\frac{\varphi_{2}^{0}}{2}=\frac{\sin\alpha\sin\varphi_{1}^{0}}{\cos\beta\sin\alpha\cos\varphi_{1}^{0}-\cos\alpha\sin\beta}, \tag{60}$$

which is the same to the third conclusion shown in equation (31).

In the first three conclusions, the motion transformation of the model is independent of the thickness *a* and hinge width *d* of the model, and only the fourth conclusion involves these two parameters. Obviously, the relationship between the dihedral angles of the

panel φ_1 and φ_i, is identical to that between the dihedral angles φ_1^0 and φ_i^0 in counterpart zero-thickness origami model throughout the entire folding process. The first, second, third conclusions are same to the fifth, sixth and seventh conclusions. Although the spatial location coordinates of some points on the panels and hinges are not always the same between thick panel origami and zero-thickness origami, the relationships of dihedral angles of different sectors are always the same such as $\varphi_1 = \varphi_1^0$, $\varphi_2 = \varphi_2^0$, $\varphi_3 = \varphi_3^0$ and $\varphi_4 + \varphi_5 = 2\varphi_4^0$. The conclusions of dihedral angles between zero-thickness and thickened four-crease single vertex origami are summarized in Supplementary Table 1.

Supplementary Table 1. Relationships of the dihedral angles between zero-thickness and thickened single-vertex four-crease origami model.

Thickened origami model	Zero-thickness origami model
$\tan\frac{\varphi_2}{2} = \frac{\sin\alpha\sin\varphi_1}{\cos\beta\sin\alpha\cos\varphi_1 - \cos\alpha\sin\beta}$	$\tan\frac{\varphi_2^0}{2} = \frac{\sin\alpha\sin\varphi_1^0}{\cos\beta\sin\alpha\cos\varphi_1^0 - \cos\alpha\sin\beta}$
$\varphi_1 = \varphi_3$	$\varphi_1^0 = \varphi_3^0$
$\cos\left(\varphi_4 + \varphi_5\right) = 1 + \frac{\sin^2\beta}{\sin^2\alpha}\left(\cos\varphi_2 - 1\right)$	$\cos(2\varphi_4^0) = 1 + \frac{\sin^2\beta}{\sin^2\alpha}(\cos\varphi_2^0 - 1)$

Supplementary Note 7. Cyclic test on self-locking thick-panel origami structure with P2P deformation mode.

As shown in Supplementary Figure 7, we performed a cyclic compression test on the self-locking thick-panel origami structure using MTS machine (10 kN load cell, USA). The self-locking thick-panel origami structure with 2 × 2 units was placed on platform (Supplementary Figure 7c), and we set this position as the initial compression position

with a loading rate of 2 mm·min^{-1}. As shown in Supplementary Fig.7b, the maximum compressive displacement was 6.95 mm (40% compressive strain), and significant stretching of the soft hinges at the bottom of the self-locking origami structure could be found. In the cyclic test, the peak force was stable for each cycle and no significant force drop was observed (as shown in Supplementary Fig.7a).

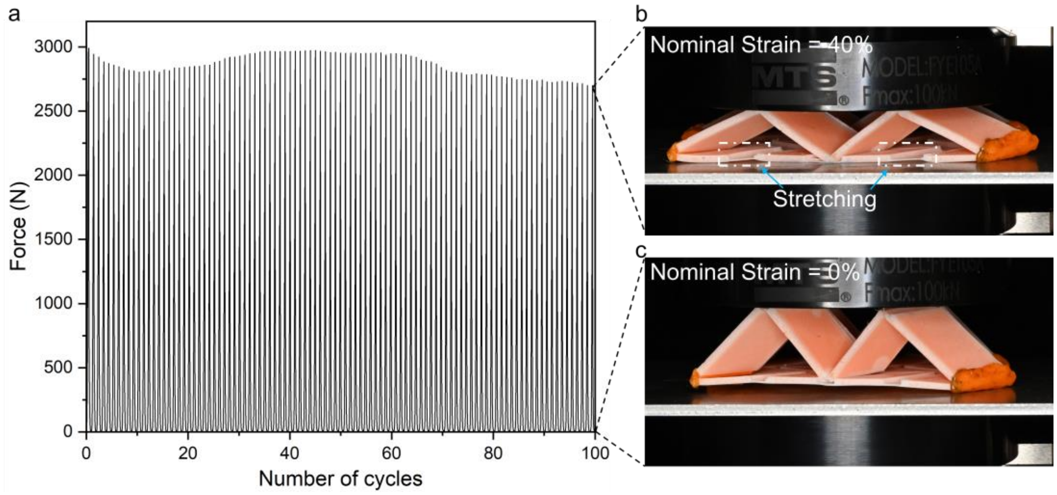

Supplementary Figure 7. Cyclic compression test for a self-locking thick-panel origami structure with 2 × 2 units. (a) Force versus number of cycles during compression. (b) Snapshot of the self-locking origami structure with 40% compressive nominal strain. (c) Snapshot of the self-locking origami structure with 0% compressive nominal strain.

Supplementary Note 8. Force analysis of self-locking thick-panel origami unit.

Supplementary Figure 8a presents geometric details of the self-locking thick panel origami unit where the upper part consists of four identical parallelograms with sides a and b as well as acute angle γ. The dihedral folding angle between the upper parallelograms and the bottom xy plane is θ ($\theta \in [0, \pi/2]$). Supplementary Figure 7b shows the cross-section of the self-locking thick-panel origami unit where the wall thickness of origami unit is t_{wall}, the length of the soft hinge is 2δ, and the initial angle

between the top and bottom panels is φ.

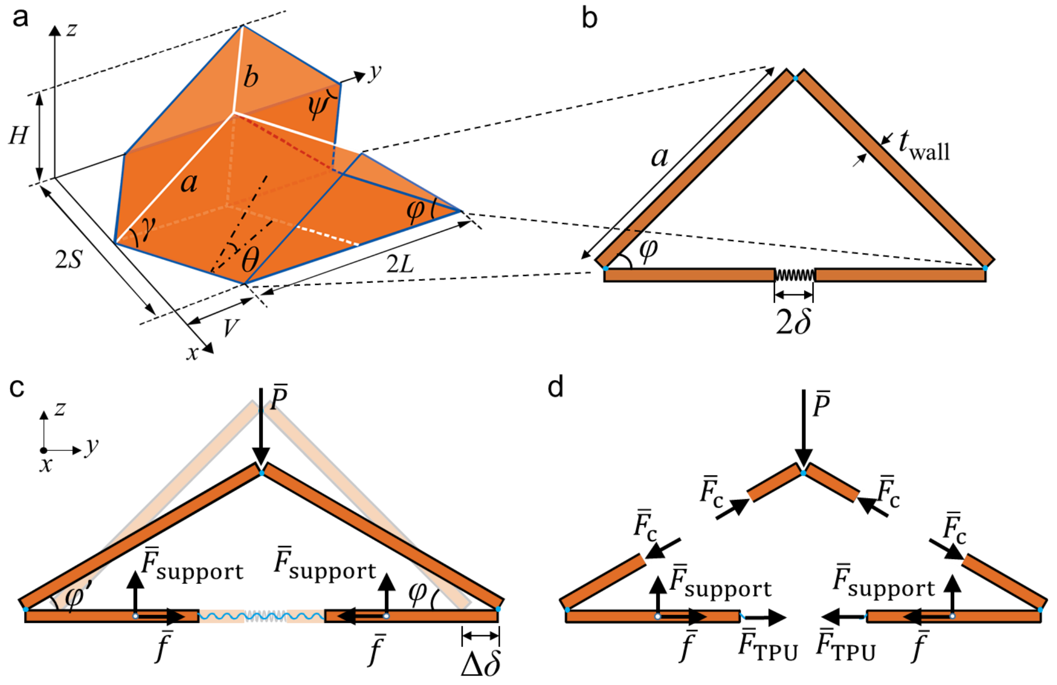

Supplementary Figure 8. Geometric model and force analysis of self-locking thick-panel origami unit. (a) Geometric model of self-locking origami unit. (b) The section view of self-locking thick-panel origami unit. (c) Force analysis of the section of self-locking thick-panel origami unit. (d) Free body diagram of the section of self-locking thick-panel origami unit.

Supplementary Figure 8a shows the geometry of the self-locking origami unit, and the relationship between these parameters can be obtained as following. The height H can be derived by $H = a \cdot \sin\gamma \cdot \sin\theta$, or $H = a \cdot \sin\varphi$, then we have:

$$\sin\gamma \cdot \sin\theta = \sin\varphi\,. \tag{61}$$

The tangent of ψ can be expressed as $\tan\psi = (a \cdot \sin\gamma \cdot \cos\theta)/(a \cdot \cos\gamma)$, then we have:

$$\tan\psi = \tan\gamma \cdot \cos\theta. \tag{62}$$

As shown in Supplementary Figure 8c, when a force P applied to the top corner of the origami unit, the applied distributed force along the top ridge is:

$$\bar{P} = P/(2b). \tag{63}$$

As the total force in the z-direction is equilibrium ($\sum F_z = 0$), we have:

$$\left(2\bar{F}_{\text{support}} - \bar{P}\right) \cdot 2b = 0, \tag{64}$$

Then we get

$$\bar{F}_{\text{support}} = \frac{\bar{P}}{2}, \tag{65}$$

where $\bar{F}_{\text{support}}$ is line-distributed support force. Under compression, the bottom panels move horizontally, and the line-distributed friction force ($\bar{f}$) on each bottom panel can be calculated as

$$\bar{f} = \mu \bar{F}_{\text{support}} = \frac{\mu \bar{P}}{2}, \tag{66}$$

where μ is the coefficient parameter between the bottom panel and ground.

Based on the free body diagram of the top corner of the origami unit (Supplementary Figure 8d), the total force in the z-direction is equilibrium ($\sum F_z = 0$) and we have:

$$\left(2\bar{F}_{\text{c}} \sin\varphi' - \bar{P}\right) \cdot 2b = 0, \tag{67}$$

Then we get

$$\bar{F}_{\text{c}} = \frac{\bar{P}}{2\sin\varphi'}, \tag{68}$$

where $\bar{F}_{\text{c}}$ is the line-distributed compressive force on a top panel. As the total force Based on the free body diagram of the left bottom corner of the origami unit (Supplementary Figure 8d), the total force in the y-direction is equilibrium ($\sum F_y = 0$), and we have:

$$\left(\bar{F}_{\text{TPU}} + \bar{f} - \bar{F}_{\text{c}} \cos\varphi'\right) \cdot 2b = 0, \tag{69}$$

Then we get

$$\bar{F}_{\text{TPU}} = \bar{F}_{\text{c}} \cos\varphi' - \bar{f}, \tag{70}$$

where $\bar{F}_{\text{TPU}}$ is the line-distributed stretching force from TPU to a bottom panel. By

combining Eq. (66), Eq. (68), and Eq. (70), $\bar{F}_{\text{TPU}}$ can be calculated as:

$$\bar{F}_{\text{TPU}} = \frac{\bar{P}}{2}\left(\cot\varphi' - \mu\right). \tag{71}$$

Supplementary Note 9. Stress-Stretch Behavior of the TPU soft hinge.

As shown in Supplementary Figure 8d, the line-distributed force action on TPU is $\bar{F}_{\text{TPU}}$. Thus, we can calculate the nominal stress acting on TPU which is along the *y*-direction (Supplementary Figure 9a):

$$s_{\text{TPU}} = s_y = \frac{\bar{F}_{\text{TPU}}}{t_{\text{TPU}}} = \frac{\bar{P}}{2t_{\text{TPU}}}\left(\cot\varphi' - \mu\right). \tag{72}$$

The TPU is uniaxially stretched, and its stretch in *y*-direction can be calculated as:

$$\lambda_y = \frac{2\delta + 2\Delta\delta}{2\delta} = 1 + \frac{\Delta\delta}{\delta}. \tag{73}$$

Since the TPU is isotropic, and no force is applied to the surface either normal to *x*-direction or *z*-direction, we have

$$\lambda_x = \lambda_z. \tag{74}$$

The TPU is incompressible, thus

$$\lambda_x \lambda_y \lambda_z = 1. \tag{75}$$

By combining Eq. (73), Eq. (74), and Eq. (75), we can calculate the relation between λ_x, λ_z and λ_y, $\Delta\delta$:

$$\lambda_x = \lambda_z = \frac{1}{\sqrt{\lambda_y}} = \frac{1}{\sqrt{1+\Delta\delta/\delta}}. \tag{76}$$

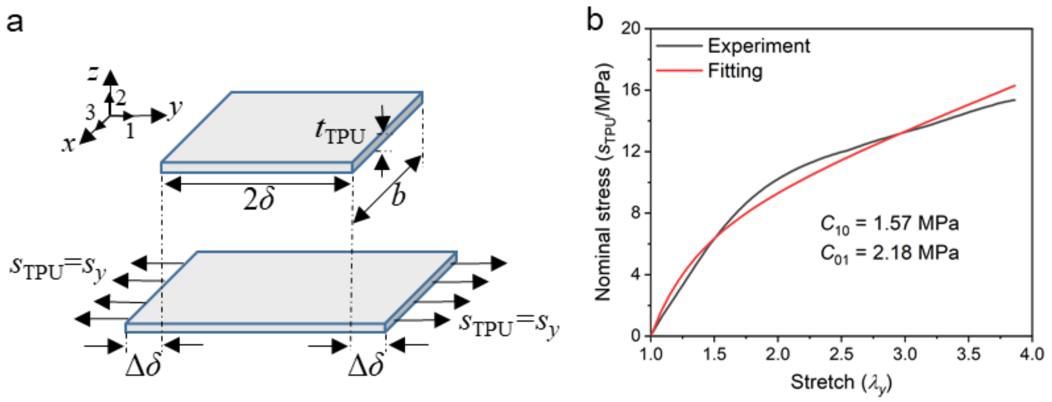

Supplementary Figure 9. Illustration of the deformation behavior of TPU. (a) Demonstration of the soft hinge (TPU) under stretching. (b) Fitting results and parameters of TPU with Mooney-Rivlin model.

Supplementary Figure 9b presents the nominal stress-stretch behavior of a TPU sample under uniaxial tension which can be captured by Mooney-Rivlin model with strain energy density function as

$$W = C_{10}(I_1 - 3) + C_{01}(I_2 - 3) + \frac{1}{D_1}(J-1)^2, \quad (77)$$

where I_1, I_2, I_3 ($I_3 = J$) are the three invariants which can be calculated using the stretches in the three principal directions (λ_1, λ_2, λ_3):

$$I_1 = \lambda_1^2 + \lambda_2^2 + \lambda_3^2, I_2 = \frac{1}{\lambda_1^2} + \frac{1}{\lambda_2^2} + \frac{1}{\lambda_3^2}, I_3 = J = \lambda_1\lambda_2\lambda_3. \quad (78)$$

For consistency with linear elasticity in the limit of small strains, it is necessary that

$$G = 2(C_{10} + C_{01}), \kappa = \frac{2}{D_1}, \quad (79)$$

where κ is the bulk modulus and G is the shear modulus.

Each principal Cauchy stress can be related directly to the partial derivative of strain energy through

$$\sigma_i = \lambda_i \frac{\partial W}{\partial \lambda_i}, \quad (80)$$

Then we get

$$\sigma_i = \lambda_i \left(\frac{\partial W}{\partial I_1} \frac{\partial I_1}{\partial \lambda_i} + \frac{\partial W}{\partial I_2} \frac{\partial I_2}{\partial \lambda_i} + \frac{\partial W}{\partial J} \frac{\partial J}{\partial \lambda_i} \right). \tag{81}$$

In particular, as shown in Supplementary Figure 9a, we set $\lambda_1 = \lambda_y$, $\lambda_2 = \lambda_z$, $\lambda_3 = \lambda_x$. So that the Cauchy stress in the stretching direction is $\sigma_1 = \sigma_y$:

$$\sigma_1 = \sigma_y = \lambda_1 \left(\frac{\partial W}{\partial I_1} \frac{\partial I_1}{\partial \lambda_1} + \frac{\partial W}{\partial I_2} \frac{\partial I_2}{\partial \lambda_1} + \frac{\partial W}{\partial J} \frac{\partial J}{\partial \lambda_1} \right)$$

$$= 2C_{10}\lambda_1^2 - 2C_{01}\frac{1}{\lambda_1^2} + \frac{2}{D_1}(J-1)\lambda_1\lambda_2\lambda_3 = 2C_{10}\lambda_y^2 - 2C_{01}\frac{1}{\lambda_y^2} + \frac{2}{D_1}(J-1)\lambda_x\lambda_y\lambda_z. \tag{82}$$

The Cauchy stresses in the other two principal directions are zero, so we have

$$\sigma_2 = \sigma_z = 2C_{10}\lambda_z^2 - 2C_{01}\frac{1}{\lambda_z^2} + \frac{2}{D_1}(J-1)\lambda_x\lambda_y\lambda_z = 0, \tag{83}$$

$$\sigma_3 = \sigma_x = 2C_{10}\lambda_x^2 - 2C_{01}\frac{1}{\lambda_x^2} + \frac{2}{D_1}(J-1)\lambda_x\lambda_y\lambda_z = 0. \tag{84}$$

To eliminating the stress contribution from volumetric change, we can subtract Eq. (83) from Eq. (82):

$$\sigma_y - \sigma_z = 2C_{10}\left(\lambda_y^2 - \lambda_z^2\right) - 2C_{01}\left(\frac{1}{\lambda_y^2} - \frac{1}{\lambda_z^2}\right). \tag{85}$$

Since $\sigma_z = 0$ and $\lambda_z = 1/\sqrt{\lambda_y}$, Eq. (85) can be rewritten to be:

$$\sigma_y = 2C_{10}\left(\lambda_y^2 - \frac{1}{\lambda_y}\right) + 2C_{01}\left(\lambda_y - \frac{1}{\lambda_y^2}\right). \tag{86}$$

Then, we can calculate the nominal stress applied to TPU:

$$s_{\mathrm{TPU}} = s_y = \frac{\sigma_y}{\lambda_y} = 2C_{10}\left(\lambda_y - \frac{1}{\lambda_y^2}\right) + 2C_{01}\left(1 - \frac{1}{\lambda_y^3}\right). \tag{87}$$

Using Eq. (87) to fit the curve in Supplementary Figure 8b, we can find that $C_{10} = 1.57\ \mathrm{MPa}$, and $C_{01} = 2.18\ \mathrm{MPa}$.

By combing Eq. (63), Eq. (72) and Eq. (87), we can build the relation between P and $\Delta\delta$:

$$P=\frac{8b\cdot t_{\mathrm{TPU}}}{\cot\varphi'-\mu}\left\{C_{10}\left[\left(1+\frac{\Delta\delta}{\delta}\right)-\left(1+\frac{\Delta\delta}{\delta}\right)^{-2}\right]+C_{01}\left[1-\left(1+\frac{\Delta\delta}{\delta}\right)^{-3}\right]\right\}. \quad (88)$$

Supplementary Note 10. Vertical displacement of top ridge of the self-locking origami unit.

As illustrated in Supplementary Figure 10, the vertical displacement ΔH of the top ridge of the origami unit mainly consists of the displacement ΔH_1 resulted from the geometric change and the displacement ΔH_2 resulted from the compression of the upper panels. Thus, we have

$$\Delta H=\Delta H_1+\Delta H_2. \quad (89)$$

Based on Supplementary Figure 10a, we can calculate the displacement ΔH_1 resulted from the geometric change:

$$\Delta H_1=a\left(\sin\varphi-\sin\varphi'\right). \quad (90)$$

In addition, based on the geometric relation, we also have that

$$a\left(\cos\varphi'-\cos\varphi\right)=\Delta\delta. \quad (91)$$

Then, based on Eq. (91), we can use $\cos\varphi$ to express $\sin\varphi'$:

$$\sin\varphi'=\frac{\sqrt{a^2-\left(\Delta\delta+a\cos\varphi\right)^2}}{a}. \quad (92)$$

So that ΔH_1 can be expressed as a function of $\Delta\delta$:

$$\Delta H_1=\Delta H_1\left(\Delta\delta\right)=a\sin\varphi-\sqrt{a^2-\left(\Delta\delta+a\cos\varphi\right)^2}. \quad (93)$$

Eq. (88) provides the relation between P and $\Delta\delta$. Thus, combining Eq. (88) and Eq. (93), we can also find that ΔH_1 is also a function of P. That is,

$$\Delta H_1=\Delta H_1\left(P\right). \quad (94)$$

Based on Supplementary Figure 10b, we can calculate the displacement ΔH_2 resulted

from the compression of the upper panels:

$$\Delta H_2 = \frac{\Delta a}{\sin\varphi'}, \tag{95}$$

where Δa is the longitudinal displacement of the upper panel caused by the compressive stress ($\sigma_c = \bar{F}_c / t_{wall}$). Thus, Δa can be calculated as:

$$\Delta a = \frac{\bar{F}_c \cdot a}{E_{panel} \cdot t_{wall}}, \tag{96}$$

where E_{panel} is Young's modulus of upper panel. Based on Eq. (63), Eq. (68), we have $\bar{F}_c = \bar{P}/2\sin\varphi'$ and $\bar{P} = P/(2b)$. Thus, Eq. (96) can be rewritten as

$$\Delta a = \frac{P \cdot a}{4\sin\varphi' \cdot E_{panel} \cdot b \cdot t_{wall}}. \tag{97}$$

Insert Eq. (97) into Eq. (95), we find that ΔH_2 is a function of *P*:

$$\Delta H_2 = \frac{P \cdot a}{4\sin\varphi \cdot \sin\varphi' \cdot E_{panel} \cdot b \cdot t_{wall}}. \tag{98}$$

Based on Eq. (89), Eq. (93) and Eq. (98), ΔH is a function of *P*:

$$\Delta H = \Delta H(P) = \Delta H_1(P) + \Delta H_2(P). \tag{99}$$

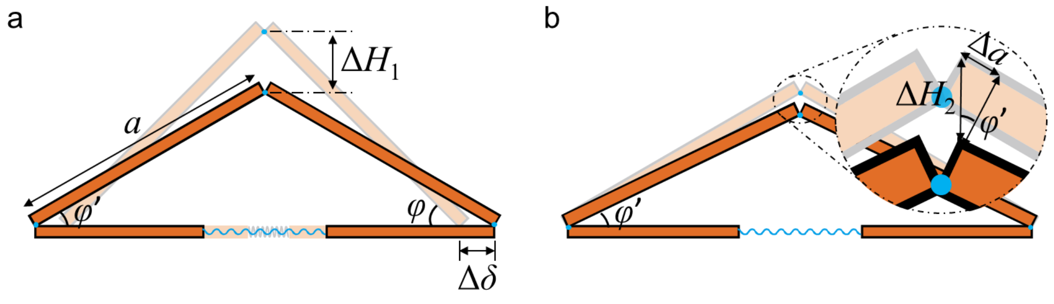

Supplementary Figure 10. (a) Illustration of the geometric change (ΔH_1) of self-locking origami unit under compression. (b) Illustration of the deformation on the wall (ΔH_2) under compression.

Supplementary Note 11. Buckling criteria for upper panels.

Above discussions are based on the assumption that the self-locking origami unit exhibits the push-to-pull (P2P) deformation mode, and the upper panels do not buckle under compression (Supplementary Figure 11a). That is, the applied force P is smaller than the critical buckling load P_{cr} that induces the buckling of upper panel (Supplementary Figure 11b). To calculate P_{cr}, we need first to calculate the critical buckling stress of upper panel σ_{cr}. We treat the upper panel as thin plate. To calculate σ_{cr}, as shown in Supplementary Figure 11c and 11d, we first consider a thin plate where all four sides are simply supported. Based on the previous work [3], the critical buckling stress $\sigma_{cr,0}$ in this case is:

$$\sigma_{cr,0} = \frac{D\pi^2}{tb^2}\left(\frac{mb}{a} + \frac{n^2 a}{b}\right)^2 . \tag{100}$$

where D is the flexural stiffness, and $D = Et^2 / \left[12\left(1-v^2\right)\right]$ with thin plate modulus E, thickness t and Poisson's ratio v. For the upper panels of the self-locking origami unit, $E = E_{panel}$, $t = t_{wall}$ and v = 0.3. *m* and *n* are the number of half-waves of buckling in the x- and y-directions, respectively. Based on experimental observations, we set that *m* = 1 and *n* = 1.

As illustrated in Supplementary Figure 11e, for the upper panel of the self-locking origami unit, only three sides are simply support. Thus, the critical buckling stress for the upper panel σ_{cr} can be calculated as

$$\sigma_{cr} = \eta \cdot \sigma_{cr,0} , \tag{101}$$

where η is the boundary condition coefficient, and set to be 0.295 (reading from the data presented in Ref. 46). Once σ_{cr} is obtained, we can find the critical buckling load:

$$P_{cr} = 4b \cdot t_{wall} \cdot \sigma_{cr} \cdot \sin\varphi' , \tag{102}$$

Geometry and material parameters used in the theoretical model were list in Supplementary Table 2.

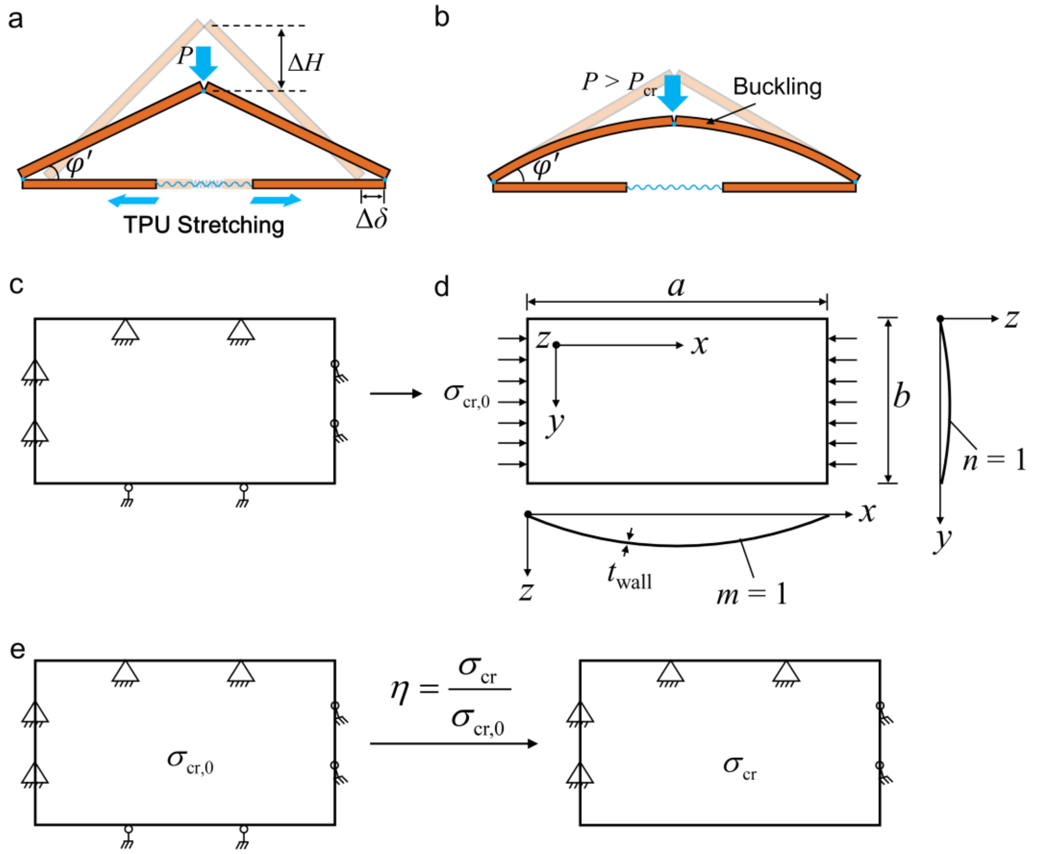

Supplementary Figure 11. Buckling criteria for upper panels of self-locking origami unit. (a) Geometric deformation under external load *P*. (b) Buckling of the unit wall if $P > P_{cr}$. (c) Thin-plate with four sides simply supported. (d) Buckling of the thin, simply supported plate under uniaxial compression. (e) The buckling stress derivation of thin plate with three sides simply supported (σ_{cr}).

Supplementary Table 2. Geometric and material parameters used in theoretic model

Geometric parameter	Value	Material parameter	Value
a	19 - 34 mm	C_{10}	1.57 MPa
φ	36 - 56 °	C_{01}	2.18 MPa
b	21.6 mm	E_{panel}	369.5 MPa
γ	60.9 °	η	0.295
t_{TPU}	0.4 mm	μ	0.3
δ	1.75 mm		

Supplementary Note 12. FEA modeling of multimaterial 3D printed thick-panel origami.

The finite element analysis (FEA) of multimaterial 3D printed thick-panel origami were conducted by using the commercially available software package ABAQUS (V6.14, Dassault Systèmes Simulia Corp., USA). In this work, we proposed the wrapping-based approach to print the rigid part of the thick-panel origami, in which the rigid panel was formed by TPU wrapped PLA. Solid tetrahedron quadratic element (element type C3D10M) was used to mesh the soft hinges and rigid panels. To accurately simulate the mechanical properties of the origami structure with low computational cost, we made following two treatments to simplify the FEA modelling. (i) In order to simplify the FEA modeling of the rigid panel formed by TPU wrapped with PLA (real design model in Supplementary Fig.12a), we treat it rigid panel as a composite (FEA model in Supplementary Fig.12a). An elastic and brittle cracking model with element deletion were implemented to capture the fracture process of composite material. Cracks were initiated when the maximum principal tensile stress exceeded 32.8 MPa. The elastic modulus of composite was set to 369.5 MPa through the curve fitting result in Supplementary Figure 12b. The hyperelastic Mooney-Rivlin model with strain energy density function $W = C_{10}(I_1 - 3) + C_{01}(I_2 - 3) + \frac{1}{D_1}(J - 1)^2$ was implement to describe the nonlinear material behavior of TPU. The material coefficients for TPU were set as C_{10}=1.57 MPa, C_{01}=2.18 MPa based on the curve fitting in Supplementary Figure 12b. (ii) To simplify the modeling of an origami structures consisting of rigid panels and soft hinges, we directly tie the soft hinges to the rigid panels. This is reasonable as in the origami printed by using the wrapping-based approach, the TPU is continuous in both rigid panel and soft hinge. As shown in Supplementary Fig.12c, we conducted uniaxial

tensile test on the RSR sample to validate this simplification. Supplementary Fig.12d compares the stress-strain curve of the RSR sample from experiment and FEA simulation, and both the simulation and experimental stress-strain curves agree well within 350% of the tensile strain.

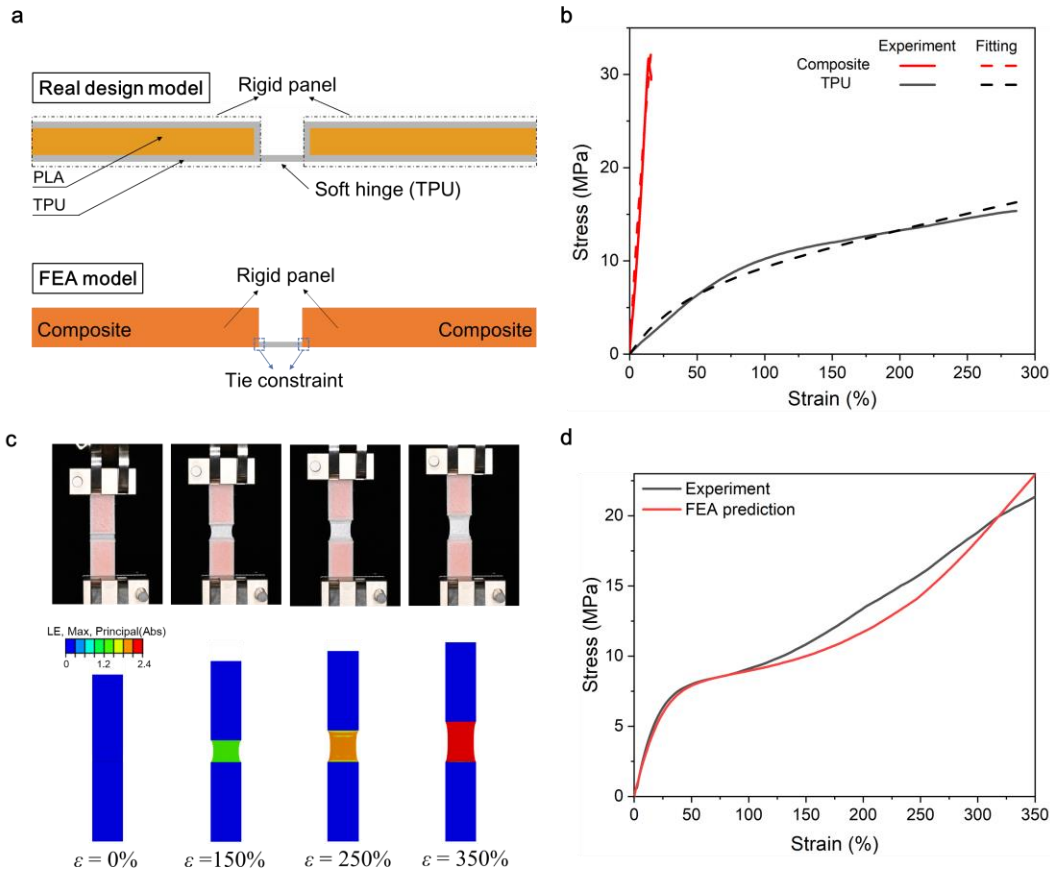

Supplementary Figure 12. Details on FEA modeling of printed thick-panel origami. (a) Illustration of the real design and FEA model of RSR (Rigid-soft-rigid) sample. (b) Fitting curve of composite material. (c) Snapshots of RSR sample during experiment and simulation. (The color scale represents the logarithmic strain (LE) of the material ranging from 0 to 2.4.) (d) Experimental and FEA prediction results of RSR sample.

Supplementary Note 13. FEA simulation of self-locking origami unit.

To explore the deformation of rigid panels and soft hinges of the self-locking origami structure during compression, FE simulations were performed for Structure I, Structure II, and Structure III in Figure 3d of the main text. Supplementary Figure 13a shows the

bottom and side views of Structure I. The arrows in Supplementary Figure 13a indicate the distribution positions of the soft hinges. Supplementary Figure 13b shows the results of the finite element analysis of Structure I during compression. We can find that the deformation occurs mainly in the soft hinges at the bottom, and the maximum tensile strain of the soft hinges exceeds 200%. Supplementary Figure 13c-13e show the stored strain energy of the three structures, which exhibit P2P, buckling, and intermediate deformation modes, respectively. It is noteworthy that structure I has the highest stored strain energy before damage (3625.7 mJ) compared to the other two structures. Moreover, the TPU soft hinges absorb 50% of the total energy as the P2P deformation mode causes the TPU soft hinges highly stretched. In contrast, the percentages of stored energy in TPU soft hinges of Structure II and Structure III are lower as the more deformations occur on the upper panels due to buckling.

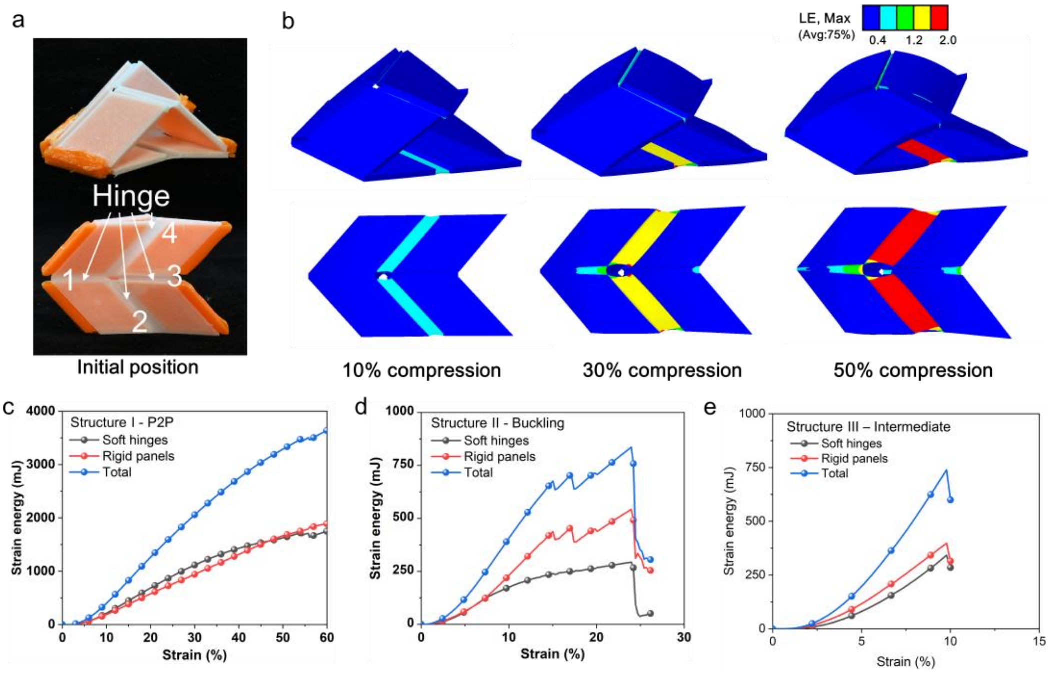

Supplementary Figure 13. FEA simulation of self-locking origami unit and the

energy efficiency analysis. (a) Bottom and side views of self-locking origami unit. (b) Snapshots of the FEA results of Structure I during compression. (The color scale represents the logarithmic strain (LE) of the material ranging from 0 to 2.) Strain energy of Structure I (c), Structure II (d), and Structure III (e) from the FEA results.

Supplementary Note 14. Fabrication of self-locking origami unit made by brass.

As shown in Supplementary Fig.14, to fabricate an origami unit with large folding angle (φ = 44°), one-step mold-pressing leads to severe factures at the folding areas (Supplementary Fig.14c). To address this issue, as shown in Supplementary Fig.14a, we need to design a series of molds with gradually increased φ, and sequentially press the brass film in Mold I to Mold III. Moreover, although the three molds can be 3D printed (Supplementary Fig.14b), to print one mold using a FDM 3D printing takes about 7.5 hours. Finally, as shown in Supplementary Figure 14d, the molded 3D origami structures need to be glued together to form the 3D origami structures.

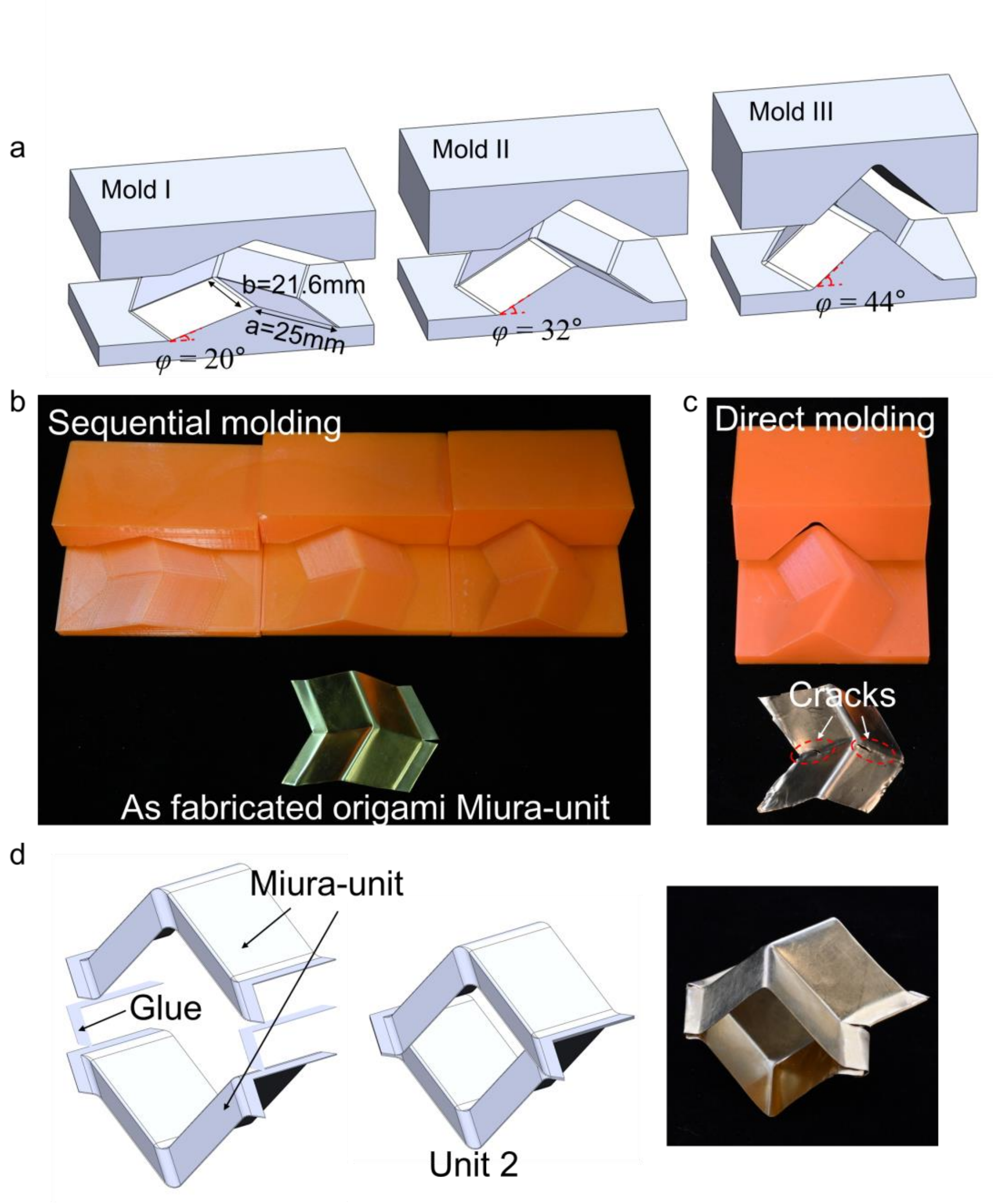

Supplementary Figure 14. Manufacture of Miura origami units made of brass by molding technology. (a) Geometric design of the molds (b) Miura origami units made through sequential molding. (c) Miura origami units made through direct molding. (d) Illustration of the assembly process of Unit 2.

Supplementary Note 15. Comparison on the energy absorption with other origami configuration.

In order to compare the energy absorption efficiency of different configurations, we performed a series of experiments to compress three different origami units. As shown

in Supplementary Fig.15a, Unit 3 is a unit cell of the self-locking origami structure (a = 25, r_t = 5, φ = 44°) proposed in this manuscript. Unit 1 has the same geometry as Unit 3, but is made of brass. Unit 2 is a unit cell designed based on the origami metamaterial configuration proposed by Ma J et al. [4], which is also fabricated from brass. The masses of the three units are similar. The compressive speed was set to 2 mm·min^{-1}.

Supplementary Figure 15b presents the compression results of the three units, where Unit 3 has the highest specific energy absorption at 50 % nominal strain ($SEA_{\varepsilon_n=50\%}$ = 0.93 J·g^{-1}), which is much higher than that of Unit 1 at 0.354 J·g^{-1} and Unit 2 at 0.07 J·g^{-1}. Besides, as shown in Supplementary Figure 15c, Unit 3 was able to recover to its initial undeformed state even after it was compressed by 50%, while Unit 1 and Unit 2 underwent plastic deformation and could not fully recover.

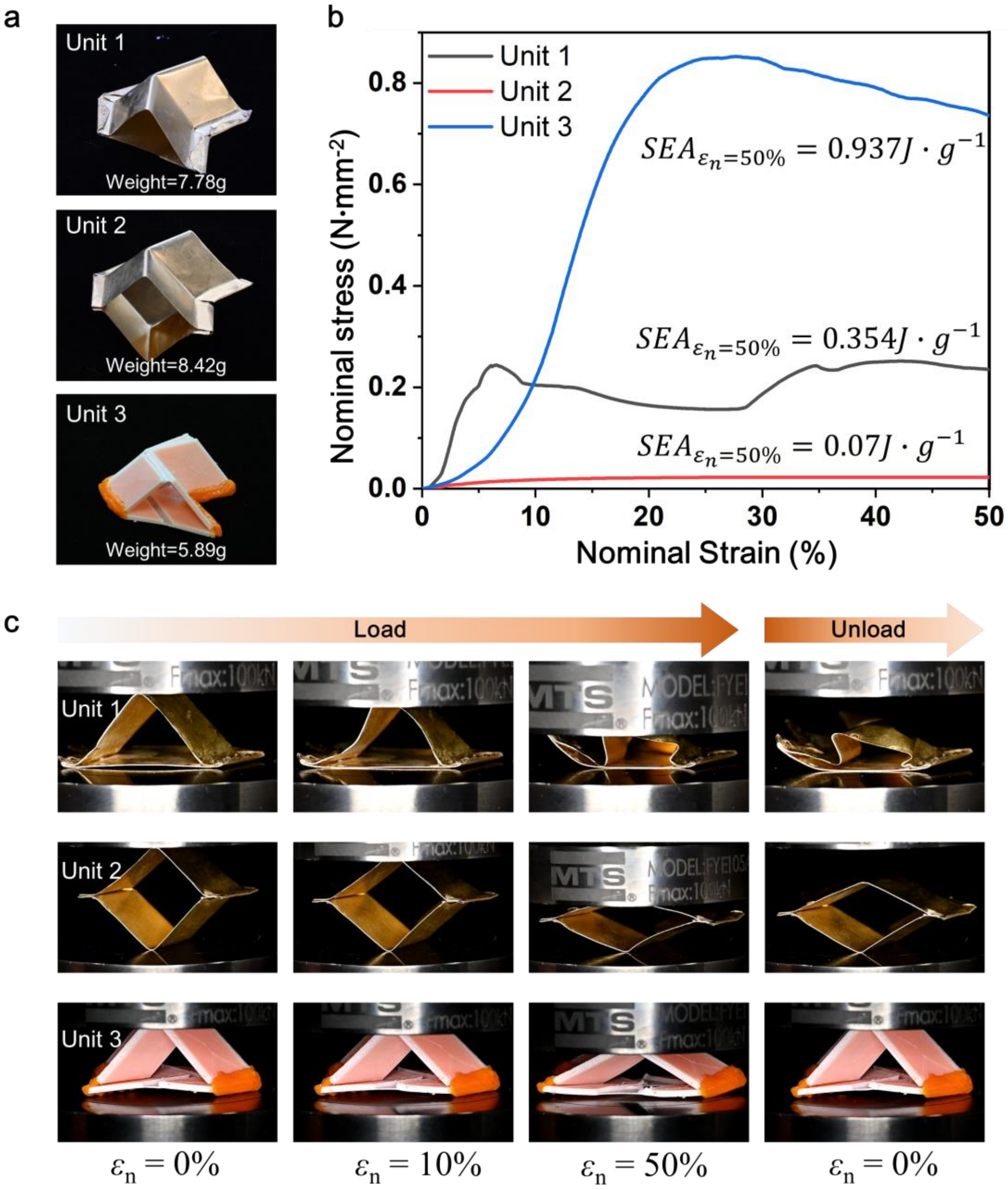

Supplementary Figure 15. Comparison of the energy absorption of Unit 1-3. (a) Image and weight of three units. (b) Compression results and specific energy absorption (SEA) of three units. (c) Snapshots of the compression process of three units.

Supplementary Note 16. Compression tests of three representative structures exhibiting three different deformation modes.

In Supplementary Figure 16, we show the loading-unloading processes for three structures (mentioned in Figure 3d of the main text) exhibiting P2P (Structure I),

buckling (Structure II), and intermediate (Structure III) deformation modes, respectively. The load and unload speed were set to 2 mm·min^{-1}. Geometry parameters of the three structures were shown in Supplementary Table 3.

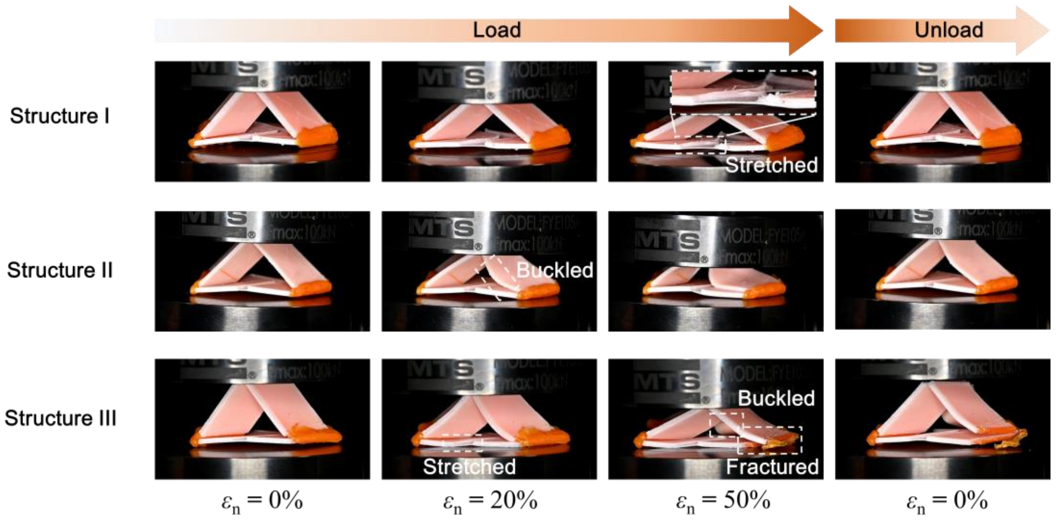

Supplementary Figure 16. Snapshots of Structure I, Structure II, and Structure III during loading-unloading.

Supplementary Table 3. Geometry parameters of Structure I - III

	a (mm)	r_t	φ (°)	H (mm)	Projection Area (mm^{2})
Structure I	25	5	44	17.37	1000.1
Structure II	25	4	44	17.37	1000.1
Structure III	25	5	52	19.7	815.7

Supplementary Note 17. Compressive strength prediction of self-locking thick-panel origami models with P2P deformation mode.

As shown in Supplementary Figure 17a, the compressive strength has been significantly improved as the increase of both thickness ratio r_t and angle φ, ranging from 0.2 N·mm^{-}

^{2} to 2 N·mm^{-2}. In Figure 17b, larger thickness ratio r_t would lead to structural strength enhancement, while increasing wall length *a* would weaken the self-locking thick-panel origami structure.

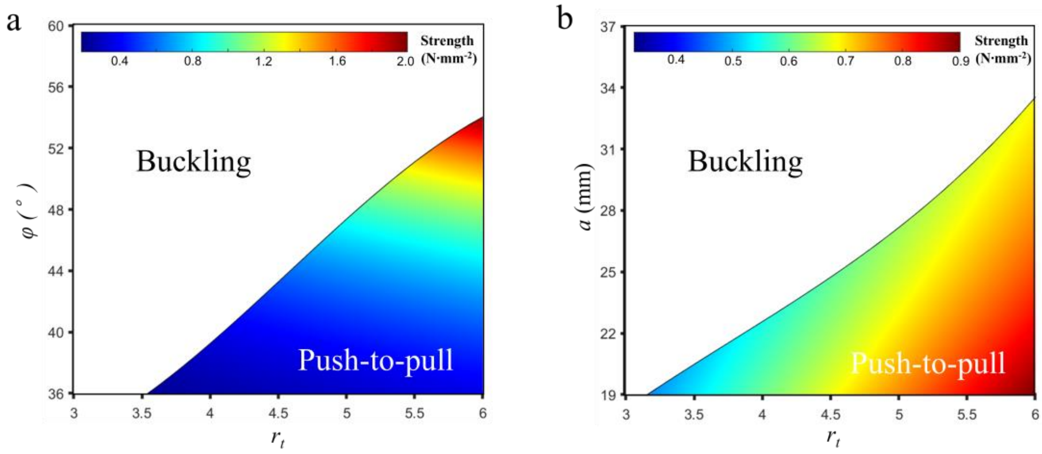

Supplementary Figure 17. Compressive strength prediction of self-locking thick-panel origami units with P2P deformation mode. (a) Compressive strength of self-locking thick-panel origami models when *a* = 25 mm. (b) Compressive strength of self-locking thick-panel origami models when φ = 44 °.

Supplementary Note 18. Flexibility and adaptability of MSO structure.

The multi-material 3D printing based origami structure fabrication approach proposed in this work gives it a certain degree of flexibility and adaptiveness (Fig. 4f in manuscript), which opens up a range of application possibilities, such as for the fabrication of lightweight and high energy absorbing helmets, as shown in Supplementary Figure 18.

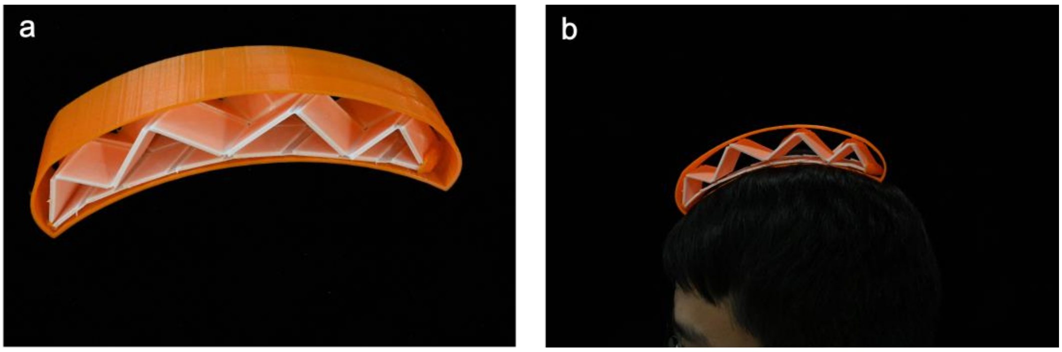

Supplementary Figure 18. Demonstrate of using MSO structure to make a safety helmet. (a) Schematic diagram of a helmet model made from a 3D printed origami structure. (b) The helmet adapts well to the curved surface of head.

Supplementary Note 19. Impact test set-up.

As shown in Supplementary Figure 19, origami structure is placed on the platform when conducting the impact test. The impact energy is calculated as *mgh*, and can be adjusted through tuning the drop height ($h \in [0, 1.8]$ m) or weight ($m \in [3.65, 32]$ kg). To acquire the response during the impact process, a force sensor is installed below the platform, whose load cell is 20000 N. The high-speed camera is used to capture the deformation behavior of thick-panel origami structures, and the high-power light source ensures that the camera can shoot clearly at high frame rates.

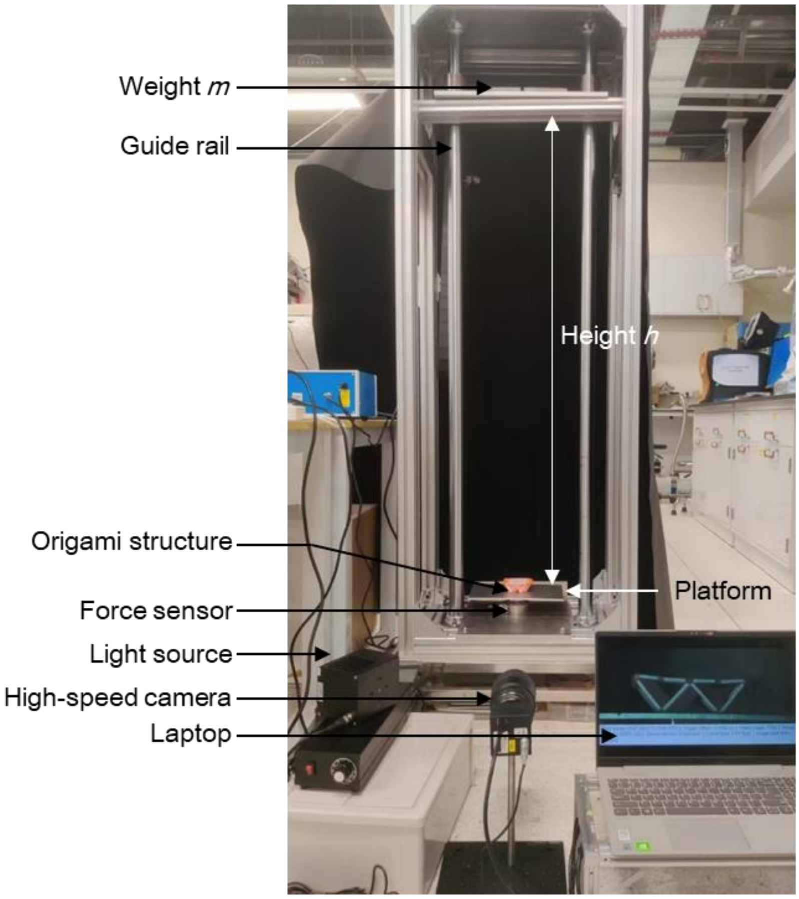

Supplementary Figure 19. Demonstration of the self-built drop tower.

Supplementary Note 20. Impact energy absorption of brass-made origami structures.

There are two key indicators to evaluate a structure's capability of absorbing impact energy [5]: (i) the value of the first peak impact force; (ii) whether there is a secondary impact. In order to demonstrate that the developed self-locking origami structures exhibit better capability of absorbing impact energy than other configurations, as shown in Supplementary Figure 20a, we fabricated two Miura-origami structures made of brass. Sample 1 has the same geometry as the developed self-locking origami structure

(Sample 3). Sample 2 was designed based on the origami metamaterial configuration proposed by Ma J et al. [4]. The weights of the three origami structures are close.

From the experimental results (Supplementary Fig.20b), the peak impact force of Sample 2 (7780.6 N) was slightly lower than that of the control group (without origami structure) under the same impact energy (24 J), and it underwent multiple secondary impacts. The peak impact force of Sample 1 (5104.1 N) was much lower than that of the control group, but it still had secondary impacts, which means that the impact energy was partially absorbed. In contrast, our 3D printed self-locking origami structure with push-to-pull mechanism not only has the largest reduction in peak force (more than 50%, 4128.9 N) but also has no significant secondary impact.

As shown in Supplementary Figure 20a, after the impact test, Sample 1 was flattened, and a large number of folds were generated due to the plastic deformation of the structures that partially absorbed the impact energy. Sample 2 was flatten, and only a few folds can be seen indicating few impact energy was absorbed. In Sample 3, the soft hinges were partially fracture, which indicates that the push-to-pull mechanism of the 3D printed origami structure was fully functional during the impact, effectively converting the impact energy into tensile deformation of the soft hinges in the bottom part, and finally dissipating the entire impact energy through the fracturing of the soft hinges.

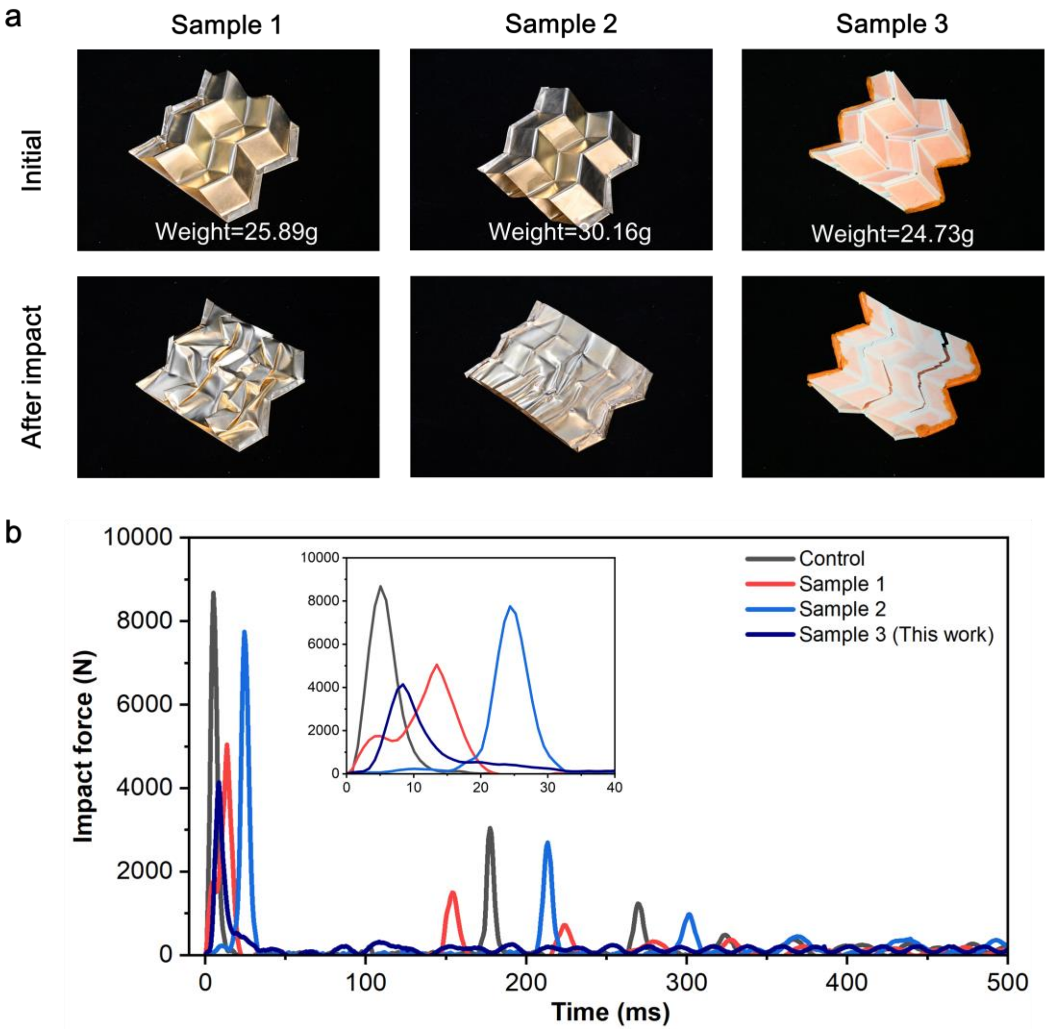

Supplementary Figure 20. Comparison of the impact energy absorption with brass-made origami structures. (a) Image of three samples before and after impact. (b) Impact response of samples at an impact energy of 24J.

Supplementary References:

[1]. Chen Y, Peng R, You Z. Origami of thick panels. *Science* **349**, 396-400 (2015).

[2]. Gu Y, Wei G, Chen Y. Thick-panel Origami Cube. *Mechanism and Machine Theory* **164** (2021).

[3] Rees DWA. Appendix B: Plate Buckling Under Uniaxial Compression. In: *Mechanics of Optimal Structural Design*) (2009).

[4] Ma J, Song J, Chen Y. An origami-inspired structure with graded stiffness.

International Journal of Mechanical Sciences **136**, 134-142 (2018).

[5] Lu G, Yu T. *Energy absorption of structures and materials*. Elsevier (2003).
