# Supplementary material for: Multimaterial 3D printed self-locking thick-panel origami metamaterials
Source: Nat Commun. 2023 Mar 23;14:1607. doi: 10.1038/s41467-023-37343-w (PMC10036479; doi:10.1038/s41467-023-37343-w)
Supplement: Supplementary file 2 — Description of Additional Supplementary Files [file 41467_2023_37343_MOESM2_ESM.pdf]

Description of Additional Supplementary Files

File name: Supplementary Movie 1
Description: the 3D printed self-locking origami structure with one unit and 2×2 units can be folded under in-plane load.

File name: Supplementary Movie 2
Description: the 3D printed self-locking origami structure can be compressed more than 100 times without significant damage under cyclic compression at a compressive strain of 40%.

File name: Supplementary Movie 3
Description: the 3D printed MSO Structure I shows a consistent push-to-pull deformation mode during compression.

File name: Supplementary Movie 4
Description: the 3D printed MSO Structure II undergoes buckling deformation of units 1 and 2 sequentially in compression, while unit 3 follows a push-to-pull deformation mode.

File name: Supplementary Movie 5
Description: when the 3D printed MSO Structure III is compressed, unit 2 is deformed first, and then, unit 3 and unit 1 are compressed sequentially. They all deform in a push-to-pull deformation mode.

File name: Supplementary Movie 6
Description: the 3D printed MSO Structure IV exhibits consistent push-to-pull deformation mode when placed on a curved platform.

File name: Supplementary Movie 7
Description: the 3D printed Impact Structure III exhibits a buckling deformation mode at an impact energy of 24 J with significant secondary impacts.

File name: Supplementary Movie 8
Description: the 3D printed Impact Structure IV exhibits a push-to-pull deformation mode at an impact energy of 24 J and absorbed the impact energy significantly.
